# Biodiversity of genes encoding anti-microbial traits within plant associated microbes

**DOI:** 10.3389/fpls.2015.00231

**Published:** 2015-04-10

**Authors:** Walaa K. Mousa, Manish N. Raizada

**Affiliations:** ^1^Department of Plant Agriculture, University of GuelphGuelph, ON, Canada; ^2^Department of Pharmacognosy, Faculty of Pharmacy, Mansoura UniversityMansoura, Egypt

**Keywords:** genes, biodiversity, evolution, plant associated microbes, rhizosphere, endophyte, antimicrobial secondary metabolites

## Abstract

The plant is an attractive versatile home for diverse associated microbes. A subset of these microbes produces a diversity of anti-microbial natural products including polyketides, non-ribosomal peptides, terpenoids, heterocylic nitrogenous compounds, volatile compounds, bacteriocins, and lytic enzymes. In recent years, detailed molecular analysis has led to a better understanding of the underlying genetic mechanisms. New genomic and bioinformatic tools have permitted comparisons of orthologous genes between species, leading to predictions of the associated evolutionary mechanisms responsible for diversification at the genetic and corresponding biochemical levels. The purpose of this review is to describe the biodiversity of biosynthetic genes of plant-associated bacteria and fungi that encode selected examples of antimicrobial natural products. For each compound, the target pathogen and biochemical mode of action are described, in order to draw attention to the complexity of these phenomena. We review recent information of the underlying molecular diversity and draw lessons through comparative genomic analysis of the orthologous coding sequences (CDS). We conclude by discussing emerging themes and gaps, discuss the metabolic pathways in the context of the phylogeny and ecology of their microbial hosts, and discuss potential evolutionary mechanisms that led to the diversification of biosynthetic gene clusters.

## Introduction

The plant is an attractive versatile home for diverse microbes that can colonize internal plant tissues (endophytes), live on the surface (epiphytes) or in the soil surrounding the root system (rhizosphere microbiota) (Barea et al., 2005; Johnston-Monje and Raizada, 2011). Plant associated microbes have the potential to be used as biocontrol, the use of living organisms to suppress crop disease (Eilenberg, 2006) through various mechanisms including the production of antibiotics (Compant et al., 2005). Diverse classes of antimicrobial secondary metabolites of microbial origin have been reported (Mousa and Raizada, 2013), including polyketides, non-ribosomal peptides, terpenoids, heterocylic nitrogenous compounds, volatile compounds, bacteriocins as well as lytic enzymes. Polyketides and non-ribosomal peptides constitute the majority of microbial derived natural products (Cane, 1997). Interestingly, the tremendous structural diversity of antimicrobial secondary metabolites originated via limited metabolic pathways utilizing few primary metabolites as precursors (Keller et al., 2005). Underlying the diversification of antimicrobial metabolites must have been a corresponding genetic diversification of ancestral genes driven by co-evolutionary pressures (Vining, 1992).

The revolution in genomics, genome mining tools and bioinformatics offers a new opportunity to connect biochemical diversity to the underlying genetic diversity and to analyze the evolutionary events leading to biodiversity (Zotchev et al., 2012; Scheffler et al., 2013; Deane and Mitchell, 2014).

The scope of this review is to describe the biodiversity of biosynthetic coding sequences (CDS) of plant-associated microbes (bacteria and fungi) that encode selected examples of antimicrobial secondary metabolites and lytic enzymes. For each example, the target pathogen(s) and mode of action are described where known, in order to highlight the diversity of biochemical targets. Out of necessity, the review focuses on compounds for which in depth molecular analysis has been conducted. We review data pertaining to the underlying molecular diversity and highlight comparative genomic data of the orthologous genes. The review concludes with a discussion of common themes and gaps in the literature, and discusses the role of evolution in the diversification of biosynthetic gene clusters including horizontal gene transfer (HGT).

## Biosynthetic genes encode diverse chemical classes of anti-microbial compounds

The diversity of compounds described in this review, the underlying genes, microbes, and pathogenic targets are summarized (Tables 1, 2).

**Table 1:** **Summary of anti-microbial compounds belonging to genetic pathways that show evidence of horizontal gene transfer (HGT)**.

**Compound**	**Chemical class**	Underlying genes^*^	**Example of producing microbe**	**Best studied pathogen target**	**Significance to plant/human**	**References**
2,4-DAFG	Phenolic polyketide	*phlD* and *phlACB* operon	*Pseudomonas fluorescens*^a^	*Gaeumannomyces graminis* var. *tritici*	Disease suppressive soils of crops	McSpadden Gardener et al., 2000; Mavrodi et al., 2001
Agrocin 84	Nucleotide analog	*agn* operon	*Agrobacterium radiobacter*^b^	*Agrobacterium* sp.	Biocontrol of crown gall	Roberts and Tate, 1977; Wang et al., 1994
Chitinases (family 18)	Lytic enzymes	See text	*P. fluorescens*^a^ *Actinoplanes missouriensis Streptomyces* sp. *Bacillus subtilis Stenotrophomonas maltophilia*	*Colletotrichum falcatum Plectosporium tabacinum*	Suppression of crop diseases such as lupin root rot	Viswanathan and Samiyappan, 2001; Kobayashi et al., 2002; El-Tarabily, 2003; Quecine et al., 2008
Chitinases (family 19)	Lytic enzymes	See text	Green and purple bacteria Actinobacteria	Various fungal pathogens	Suppression of crop diseases	Prakash et al., 2010
Helvolic acid	Fusidane triterpene	See text	*Aspergillus fumigates*	*Magnaporthe oryzae*	Rice blast disease	Lodeiro et al., 2009; Zhao et al., 2010
Loline	Indole alkaloid	LOL-1 and LOL-2	Neotyphodium uncinatum^*^ Epichloe	Insects	Protects its host plants from insects	Blankenship et al., 2001; Schardl, 2010
Mupirocin	Polyketide	*mup* operon	*P. fluorescens*^a^	Methicillin resistant *Staphylococci* and *Streptococci Haemophilus influenzae Neisseria gonorrheae*	Clinical use for human	Fuller et al., 1971; Sutherland et al., 1985; Whatling et al., 1995; Gurney and Thomas, 2011
Paclitaxel	Diterpene	See text	*Penicillium aurantiogriseum*	*Heterobasidion annosum Phaeolus schweinitzii Perenniporia subacida*	Anticancer	Zhou et al., 2010; Soliman et al., 2013
Phenazines	Heterocyclic nitrogenous compounds	*phzADEFG* operon	*P. fluorescens*^a^^*^ *P. aeruginosa Burkholderia cepacia Pantoea agglomerans* (*Erwinia herbicola*)	*Rhizoctonia solani Gaeumannomyces graminis* var. *tritici Pythium* spp. *Fusarium oxysporum*	Combat soil borne pathogens	Ligon et al., 2000 Gurusiddaiah et al., 1986; Anjaiah et al., 1998; McDonald et al., 2001; Mavrodi et al., 2013
Pyoluteorin	Phenolic polyketide	*pltABCDEFG* operon	*P. fluorescens*^a^	*P. ultimum*	Damping-off disease in cotton	Howell and Stipanovic, 1980
Pyrrolnitrin	Chlorinated phenylpyrrole	*prnABCD* operon	*P. fluorescens*^a^^*^ *Br. pyrrocinia Myxococcus fulvus Pantoea agglomerans Serratia* sp.	*Trichophyton* sp. *Botrytis cinerea Rhizoctonia solani G. graminis* var. *tritic*	Treatment of skin mycoses	Arima et al., 1964; Hammer et al., 1993; Chernin et al., 1996; El-Banna and Winkelmann, 1998; Kirner et al., 1998; Hammer et al., 1999; Tazawa et al., 2000
Zwittermicin A	Polyketide/nonribosomal peptide hybrid	*zmaA- zmaV* and *kabR*, *kabA—kabD*	*B. cereus*^c^^*^ *B. thuringiensis*	*Phytophthora medicaginis*	Contro root rot in alfalfa and chickpeas	Raffel et al., 1996; Silo-Suh et al., 1998; Kevany et al., 2009

* *Indicates that the genes noted have been studied in this species*.

A superscript beside the species refers to the recent genome sequence data as indicated below;

a P. fluorescens (Martínez-García et al., 2015)

b Agrobacterium radiobacter (Zhang et al., 2014)

c *B. cereus (Takeno et al., 2012)*.

**Table 2:** **Summary of anti-microbial compounds belonging to genetic pathways, grouped by different evolutionary levels of diversification**.

**Compound**	**Chemical class**	Underlying genes^*^	**Example of producing microbe**	**Best studied pathogen target**	**Significance to plant/human**	**References**
**GENOME LEVEL DIVERSIFICATION**						
Difficidin	Polyketide	PKS3	*B. amyloliquefaciens*^a^^*^ *B. subtilis*	Broad spectrum antibacterial	Crop and human pathogens	Zimmerman et al., 1987; Chen et al., 2006, 2009.
Loline	Indole alkaloid	LOL-1 and LOL-2	Neotyphodium uncinatum^*^ Epichloe	Insects	Protects its host plants from insects	Blankenship et al., 2001; Schardl, 2010
**INTRA GENE CLUSTER DIVERSIFICATION**						
Agrocin 84	Nucleotide analog	*agn* operon	*Agrobacterium radiobacter*^b^	*Agrobacterium* sp.	Biocontrol of crown gall	Roberts and Tate, 1977; Wang et al., 1994
Polymyxins A, B, D, E (colistin), and M (mattacin)	Non-ribosomal lipopeptide	*pmxA-E* operon	*Paenibacillus polymyxa*^c^	*Klebsiella pneumonia P. aeruginosa Acinetobacter* sp.	Limited clinical application due to toxicity	Storm et al., 1977; Gales et al., 2006; Li et al., 2006; Shaheen et al., 2011.
Trichodermin and Harzianum A	Terpenoids	*tri* cluster	Trichoderma arundinaceum^*^ T. brevicompactum^*^	*Rhizoctonia solani Alternaria solani*	Crop pathogens	Chen et al., 2007; Cardoza et al., 2011; Tijerino et al., 2011
**DIVERSIFICATION WITHIN CODING SEQUENCES (CDS)**						
Chitinases (family 18)	Lytic enzymes	See text	*P. fluorescens*^d^ *Actinoplanes missouriensis Streptomyces* sp. *Bacillus* sp. *Stenotrophomonas maltophilia*	*Colletotrichum falcatum Plectosporium tabacinum*	Suppression of crop diseases such as lupin root rot	Viswanathan and Samiyappan, 2001; Kobayashi et al., 2002; El-Tarabily, 2003; Quecine et al., 2008
Ergots	Alkaloid	See text	*Epichloe Neotyphodium Aspergillus* sp. *A. fumigatus Penicillium* sp. *Claviceps purpurea*	Nematodes, insects, and mammalian herbivores	Protect the host plant	de Groot et al., 1998; Gröger and Floss, 1998; Schardl, 2010
Fusaricidins	Non-ribosomal peptides	*fusGFEDCBA* operon	*P*. *polymyxa*^c^	*Aspergillus niger Aspergillus oryzae Fusarium oxysporum Penicillium thomii Leptosphaeria maculans*	Control black root rot in canola	Kajimura and Kaneda, 1996, 1997; Beatty and Jensen, 2002; Schwarzer et al., 2003
Iturins bacillomycins D, F and L, bacillopeptins, iturins A, C, E and E, and mycosubtilins	Non-ribosomal cyclolipopeptides	*ituDABC* operon	B. subtilis^e^^*^ B. amyloliquefaciens^a^^*^	*Rhizoctonia solani Fusarium oxysporum F. graminearum*	Targets Gibberella ear rot	Gueldner et al., 1988; Constantinescu, 2001; Tsuge et al., 2001; Leclère et al., 2005; Dunlap et al., 2013; Hamdache et al., 2013
**COMPLEX DYNAMIC INTERACTION**						
Jadomycin	Angucycline polyketide	*jad* operon	*Streptomyces venezuelae*	Methicillin-resistant *S. aureus*	Clinical use	Doull et al., 1994; Jakeman et al., 2009
**OTHERS**						
Bacilysin	Non-ribosomal peptide	*ywfB-G* operon and *ywfH*	B. subtilis^e^^*^ B. amyloliquefaciens B. pumilus	*Candida albicans*	Clinical use	Kenig and Abraham, 1976; Leoffler et al., 1986; Phister et al., 2004
HCN	Volatile compound	*hcnABC* operon	P. aeruginosa^*^ P. fluorescens	*Thielaviopsis basicola*	Black root rot of tobacco	Voisard et al., 1989, 1994; Frapolli et al., 2012
Phomenone	Sesquiterpene	See text	*Xylaria* sp.	*Cladosporium cladosporioides*	Targets fungal wheat pathogen	Silva et al., 2010

* *Indicates that the genes noted have been studied in this species*.

A superscript beside the species refers to the recent genome sequence data as indicated below

a *B. amyloliquefaciens (Kim et al., 2015)*,

b *Agrobacterium radiobacter (Zhang et al., 2014)*,

c *P. polymyxa (Xie et al., 2014)*,

d *P. fluorescens (Martínez-García et al., 2015)*,

e *B. subtilis (Barbe et al., 2009; Belda et al., 2013)*.

### Polyketides

**Figure 1:**
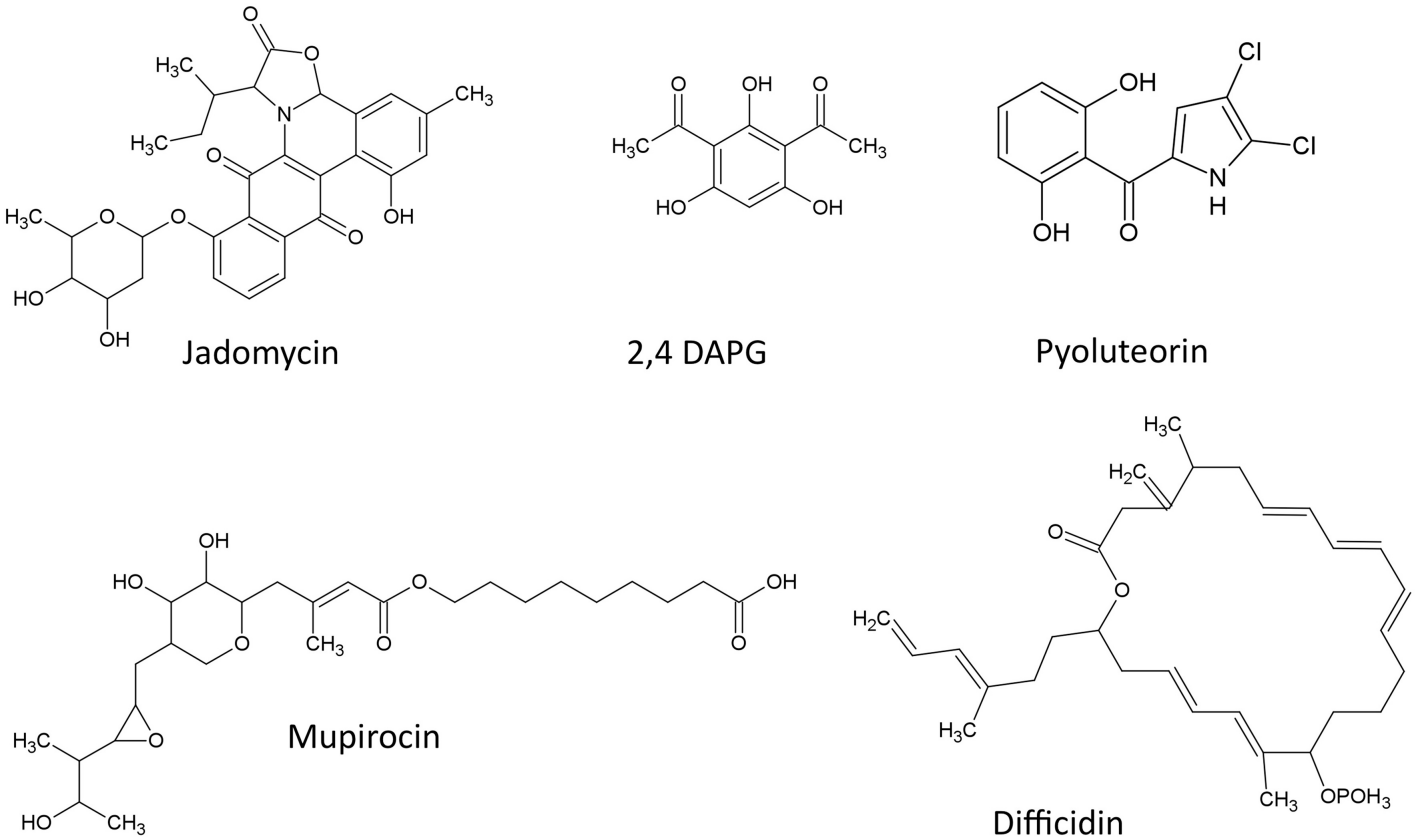
**Structures of polyketide compounds featured in this review**.

The structures of Polyketides described in this review are shown (Figure 1)

#### 2,4-diacetylphloroglucinol

2,4-diacetylphloroglucinol (2,4-DAPG) is a well-studied fluorescent polyketide metabolite produced by many strains of fluorescent *Pseudomonas* spp. that contributes to disease-suppressive soils of crops (McSpadden Gardener et al., 2000; Mavrodi et al., 2001). 2,4-DAPG is synthesized by the condensation of three molecules of acetyl coenzyme A and one molecule of malonyl coenzyme A to produce the precursor monoacetylphloroglucinol (MAPG) (Shanahan et al., 1992). In *P. fluorescens* strain Q2-87, four coding sequences (CDS) within the *phl* operon are responsible for biosynthesis of 2,4-DAPG: a single CDS (*phlD*) encoding a type III polyketide synthase is responsible for the production of phloroglucinol from the condensation of three acetyl-CoAs, and then three CDS (*phlACB*) encoding acetyltransferases are sufficient to convert phloroglucinol to 2,4-DAPG via MAPG (Bangera and Thomashow, 1999; Yang and Cao, 2012). It was suggested that the peptides encoded by *phlACB* may exist as a multi-enzyme complex (Bangera and Thomashow, 1999). *phlD* has been the subject of interest, because it has homology to chalcone and stilbene synthases from plants, which suggests horizontal gene transfer (HGT) between plants and their rhizosphere microbial populations (Bangera and Thomashow, 1999). Whereas, *phlACB* coding sequences are highly conserved between eubacteria and archaebacteria (Picard et al., 2000), a considerable degree of polymorphism was reported for *phlD* (Mavrodi et al., 2001). *phlA* transcription is negatively regulated by the product of *phlF* (Delany et al., 2000) which also appears to mediate repression by fusaric acid (Delany et al., 2000), a metabolite of pathogenic fungi of plants, that has previously been implicated in repression of biosynthesis of the anti-fungal compound, phenazine (see above) (van Rij et al., 2005). These observations demonstrate the ongoing arms race between plants, their fungal pathogens and associated anti-fungal antagonists, leading to gene diversification.

#### Mupirocin

The polyketide mupirocin or pseudomonic acid is one of the major antibacterial metabolites produced by *Pseudomonas fluorescens* (Fuller et al., 1971) and is widely used as a clinical antibiotic (Gurney and Thomas, 2011). Mupirocin can inhibit the growth of methicillin resistant *Staphylococci, Streptococci, Haemophilus influenza*, and *Neisseria gonorrheae* (Sutherland et al., 1985). In terms of the mode of action, mupirocin inhibits isoleucyl-tRNA synthetase, and hence prevents incorporation of isoleucine into newly synthesized proteins, thus terminating protein synthesis (Hughes and Mellows, 1980). Biochemically, mupirocin has a unique chemical structure that contains a C9 saturated fatty acid (9-hydroxynonanoic acid) linked to C17 monic acid A (a heptaketide) by an ester linkage (Whatling et al., 1995). Mupirocin is derived from acetate units incorporated into monic acid A and 9—hydroxynonanoic acid via polyketide synthesis (Whatling et al., 1995). At the molecular level, the mupirocin biosynthetic gene cluster (*mup* operon) in *P*. *fluorescens* is complex, and includes 6 Type I polyketide synthases that are multifunctional as well as 29 proteins of single function within a 65 kb region, which are incorporated into 6 larger coding sequences (modules *mmpA-F*) (El-Sayed et al., 2003; Gurney and Thomas, 2011). The gene cluster is non-standard as the CDS are not in the same order as the biosynthetic steps (El-Sayed et al., 2003; Gurney and Thomas, 2011). The acyltransferase (AT) domains of the polyketide synthases (PKS) are not present in each genetic module but are instead encoded by a separate CDS (from the *mmpC* module) and this classifies these PKS as *in-trans* AT PKSs (El-Sayed et al., 2003). With respect to gene regulation, two putative regulatory genes, *mupR* and *mupI*, were identified within the cluster that are involved in quorum sensing (QS) dependent regulation (El-Sayed et al., 2001).

An interesting feature of this system in *P. fluorescens* is that self-resistance to mupirocin is also encoded by a CDS (*mupM*) within the biosynthetic gene cluster (El-Sayed et al., 2003). *mupM* encodes a resistant Ile t-RNA synthetase (IleS) due to polymorphisms within the binding site of mupirocin (El-Sayed et al., 2003; Gurney and Thomas, 2011). A second resistant IleS was cloned from *P. fluorescens* NCIMB 10586 outside of the *mup* gene cluster which showed 28% similarity to the *mupM* product (Yanagisawa et al., 1994). Human pathogens that have high level mupirocin-resistance are associated with an additional gene that encode a novel IleS with similarity to eukaryotic counterparts; this resistance gene is associated with transposable elements and is carried on plasmids, facilitating its rapid spread (Eltringham, 1997; Gurney and Thomas, 2011).

There is also genetic evidence that the entire *mup* gene cluster in *Pseudomonas* arose by horizontal gene transfer; specifically the genes encoding tRNA_Val_ and tRNA_Asp_ were found upstream of the *mupA* promoter region leading to speculation that the *mup* cluster arose from homologous recombination between chromosomal tRNA genes and possibly a plasmid containing the *mup* cluster (El-Sayed et al., 2003). The inclusion of a resistant IleS (*mupM*) within the *mup* biosynthetic cluster might have facilitated such horizontal gene transfer, as otherwise uptake of the mupirocin gene cluster would have been immediately suicidal.

#### Difficidin

Difficidin is a polyketide with an interesting geometry that involves four double bonds in the Z configuration (Chen et al., 2006). Difficidin is produced by various *Bacillus species* such as *B. subtilis* and *B. amyloliquefaciens* FZB 42 with broad antibacterial activity against human and crop pathogens (Zimmerman et al., 1987; Chen et al., 2006, 2009). A large gene cluster (*pks3*) encoding difficidin (and oxydifficidin) was characterized in *B. amyloliquefaciens* (Chen et al., 2006). This compound is included in this review, because *pks3* is adjacent to other polyketide synthesis gene clusters, *pks1* and *pks2*, that encode bacillaene and macrolactin, respectively (Chen et al., 2006; Schneider et al., 2007). All three gene clusters share sequence homology, a similar order of CDS and are located close to another on the chromosome, leading Chen et al. (2006) to hypothesize that they emerged from homologous recombination from a common ancestral gene cluster resulting in gene duplication. This system provides insights into the diversification of polyketides.

#### Pyoluteorin

Pyoluteorin (PLt) is a phenolic polyketide with bactericidal, herbicidal, and fungicidal properties. Plt can suppress damping-off disease in cotton caused by the fungus, *Pythium ultimum* (Howell and Stipanovic, 1980). Both PLt and phenazine (see below) may act synergistically to suppress such soil-borne fungal diseases in plants, as some studies have suggested that the two biosynthetic pathway interact with one another (Ge et al., 2007; Lu et al., 2009). The biosynthesis of Plt involves condensation of proline with three acetate equivalents through chlorination and oxidation. The carbon skeleton is built up by the action of a single multienzyme complex (Nowak-Thompson et al., 1999). In *Pseudomonas fluorescens* Pf-5, a 24 kb segment contains the PLt biosynthetic operon (*pltABCDEFG*). PLt biosynthesis is catalyzed by type I polyketide synthases (*pltB*, *pltC*), an acyl-CoA dehydrogenase (*pltE*), an acyl-CoA synthetase (*pltF*), a thioesterase (*pltG*), and halogenases (*pltA*, *pltD, pltM*) with *pltM* located adjacent to the gene cluster (Nowak-Thompson et al., 1999). A significant delay in the expression of the PLt biosynthetic operon was reported in the cucumber spermosphere compared to cotton, which correlated to the timing of infection with the fungal root pathogen *Pythium ultimum* (Kraus and Loper, 1995). The authors suggest that such temporal differences may be responsible for differential disease suppression in diverse plant hosts.

The *plt* biosynthetic operon has been shown to be regulated by a LysR family transcriptional activator, encoded by *pltR* (Nowak-Thompson et al., 1999). Interestingly, *pltR* is tightly linked and transcribed divergently to the biosynthetic gene cluster (Nowak-Thompson et al., 1999). In earlier studies involving the biosynthetic operon of phenazine, its LysR transcriptional regulator gene (*phzR*) was also shown to be tightly linked to its corresponding biosynthesis gene cluster (Pierson et al., 1998). As both phenazine and PLt combat soil-borne fungal diseases in plants, we speculate that strong evolutionary pressures in the rhizosphere may have promoted HGT of the biosynthetic operons to new rhizosphere microbial hosts; the activator-cluster gene module would facilitate activation of the biosynthetic CDS following such gene transfer.

#### Jadomycin

Jadomycin is a member of angucycline antibiotics produced by *Streptomyces* species such as *S. venezuelae*. Jadomycin (*jad*) production is induced under stress conditions such as phage infection or heat shock (Doull et al., 1994; Jakeman et al., 2009). The *jad* biosynthetic gene cluster in *S. venezuelae* is closely related to type II polyketide synthase genes (Han et al., 1994) with a complex biosynthetic gene cluster (Zou et al., 2014). Jadomycin is of interest here because upstream of the *jad* operon are sets of negative regulatory genes including *jadR1R2R3* and *jadW123* (Yang et al., 1995; Zou et al., 2014). *jadW123* encodes enzymes for the biosynthesis of gamma-butyrolactones (GBL), whereas JadR2 is a pseudoreceptor for GBL which upon its binding activates JadR1 and JadR3 that subsequently act as positive and negative transcriptional regulators of the *jad* biosynthetic operon, respectively (Zou et al., 2014). GBLs are becoming well known as regulators of secondary metabolism in gram positive bacteria, analogous to the related acyl homoserine lactone compounds which mediate QS in gram negative bacteria (Nodwell, 2014). QS is a method of communication between bacterial populations that activates genes based on high cell density through the signal molecule N-acyl-homoserine lactone (AHL) (Whitehead et al., 2001). Whereas, QS signaling molecules are thought to be synthesized and sensed by the same species (Nodwell, 2014), the GBL/*jad* system is interesting, because recent data suggests that GBL can signal across different *Streptomyces* species to activate different polyketide biosynthetic pathways (Nodwell, 2014; Zou et al., 2014). Biologically, it has been shown that different *Streptomyces* species, which are soil microbes, can live on the same grain of soil alongside a diversity of bacteria (Keller and Surette, 2006; Vetsigian et al., 2011), suggesting there may have been evolutionary selection for inter-species coordination for antibiotic production (Nodwell, 2014), resulting in enhanced genetic complexity associated with the *jad* locus.

### Non-ribosomal peptides

**Figure 2:**
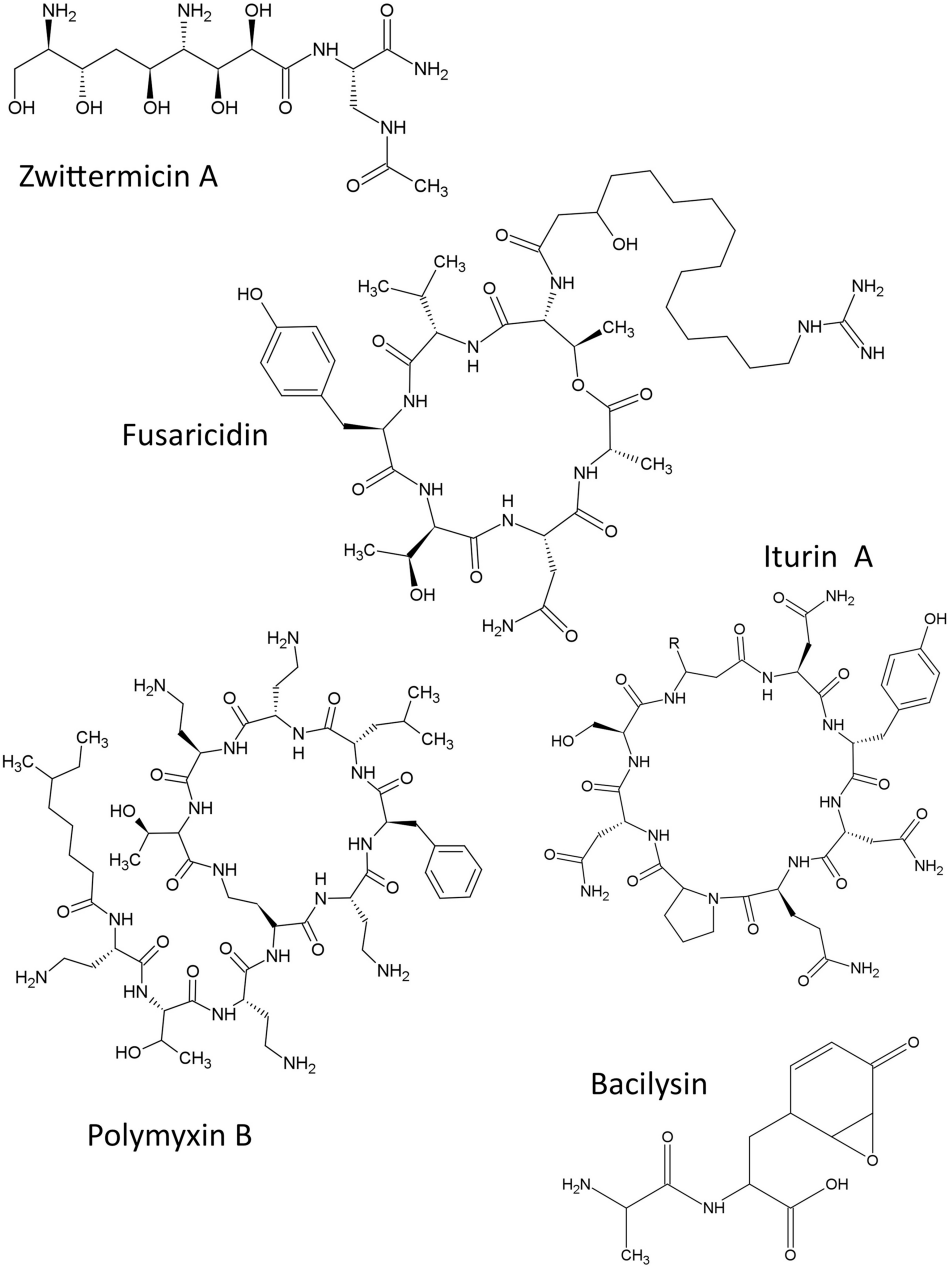
**Structures of non-ribosomal peptide compounds featured in this review**.

The structures of non-ribosomal peptides described in this review are shown (Figure 2).

#### Zwittermicin A

Zwittermicin A is a polyketide/nonribosomal peptide hybrid antibiotic produced by *B. cereus* and *B. thuringiensis* (Raffel et al., 1996) with activity against oomycetes such as *Phytophthora medicaginis* and some other pathogenic fungi (Silo-Suh et al., 1998). Zwittermicin A has a unique structure that includes glycolyl moieties, D amino acid, and ethanolamine in addition to the unusual terminal amide produced from the ureidoalanine (nonproteinogenic amino acid) (Kevany et al., 2009). Zwittermicin A is thought to be biosynthesized as part of a larger metabolite that is processed twice to form zwittermicin A and two other metabolites (Kevany et al., 2009). The complete biosynthetic operon encoding zwittermicin A includes 27 open reading frames (CDS, *zmaA*, and *zmaV*) that extend over 62.5 kb of the *Bacillus cereus* UW85 genome, in addition to five individual genes (*kabR* and *kabA—kabD*) (Kevany et al., 2009). In this study, support was gained for the hypothesis that the skeleton of zwittermicin A is catalyzed by a megasynthase enzyme involving multiple nonribosomal peptide synthetases (NRPS) and PKS; the megasynthase has multiple modules containing distinct domains that catalyze the different steps in the pathway (Emmert et al., 2004; Kevany et al., 2009). Evidence suggested that the CDS included 5 NRPS modules (Kevany et al., 2009). It is noteworthy that a similar gene cluster was characterized on a plasmid in *B. cereus* AH1134, suggesting that the pathway can be transferred horizontally (Kevany et al., 2009). Consistent with the mobility of this operon, an orthologous 72-kb region encoding for zwittermicin A in *Bacillus thuringiensis*, was shown to be flanked by putative transposase genes on both edges, suggesting that it may be a mobile element that was gained by *B. cereus* through horizontal gene transfer. Since zwittermicin A has been reported to enhance the activity of protein toxins that attack insects (Broderick et al., 2000), it was hypothesized that transfer of this operon into *B. thuringiensis* permitted the microbe to gain insecticide-promoting factors to combat insects during co-evolution (Luo et al., 2011).

#### Fusaricidins A–D

Fusaricidins are guanidinylated ß-hydroxy fatty acids attached to a cyclic hexapeptide including four D-amino acids (Kajimura and Kaneda, 1997; Schwarzer et al., 2003). These antibiotics are produced by *Paenibacillus polymyxa* strains and exhibit antifungal activity against diverse plant pathogens including, *Aspergillus niger*, *Aspergillus oryzae*, *Fusarium oxysporum*, and *Penicillium thomii* (Kajimura and Kaneda, 1996, 1997) as well as *Leptosphaeria maculans*, the causal agent of black root rot in canola (Beatty and Jensen, 2002). The amino acid chains of fusaricidins are linked together and modified by a non-ribosomal peptide synthetase (NRPS). The multi-domain NRPS consists of up to 15,000 amino acids and is therefore considered among the longest proteins in nature (Schwarzer et al., 2003). NRPS incorporation is not limited to the 21 standard amino acids translated by the ribosome, and this promiscuity contributes to the great structural diversity and biological activity of non-ribosomal peptides (Li and Jensen, 2008).

In *P*. *polymyxa* E68, the fusaricidin biosynthetic gene cluster (*fusGFEDCBA*) has been characterized in which the NRPS coding sequence, the largest CDS in the cluster, was observed to encode a six-module peptide (Choi et al., 2008; Li and Jensen, 2008; Li et al., 2013). The biosynthetic cluster includes other CDS responsible for biosynthesis of the lipid moiety but does not contain transporter genes (Li and Jensen, 2008). In *P. polymyxa*, a promoter for the *fus* operon was identified and shown to be bound by a transcriptional repressor (AbrB) which previous studies implicated as a regulator of sporulation; this is of interest since fusaricidin was observed to be synthesized during sporulation, thus coordinating the microbe's secondary metabolism with its life cycle (Li et al., 2013).

**Figure 3:**
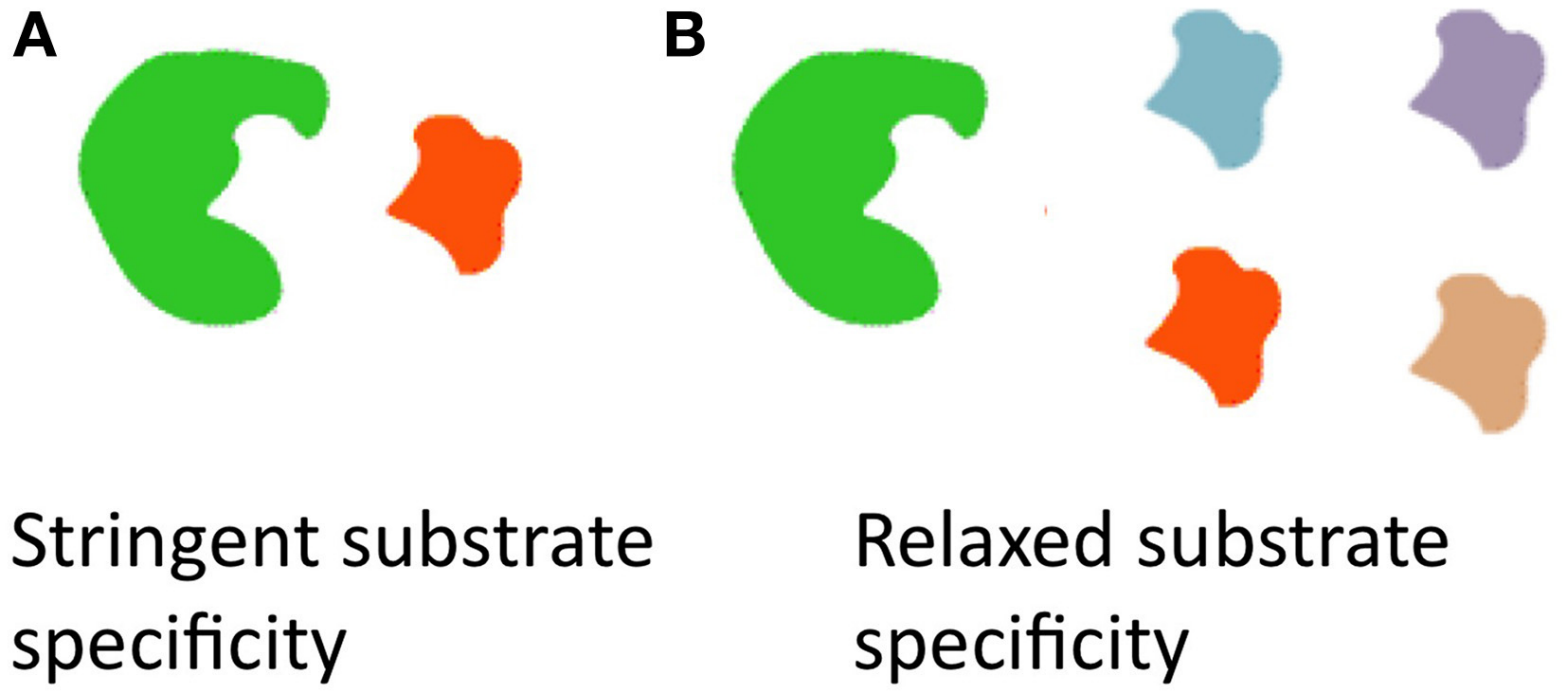
**Diagram illustrating how a diversity of fusaricidins are produced from a single allele of *fusA* which encodes the non-ribosomal peptide synthase (NRPS) A-domain. (A)** Most enzymes have stringent substrate specificity. **(B)** By contrast, the NRPS A-domain can recognize and incorporate different amino acids to create diverse fusaricidins, and hence it is an example of an enzyme with relaxed substrate specificity (Han et al., 2012).

Allelic diversity is typically thought to be responsible for producing chemical diversity. However, an interesting feature of the *fus* cluster is that a diversity of fusaricidins, differing in their incorporated amino acids (Tyr, Val, Ile, allo-Ile, Phe), can be produced by a single allele of *fusA;* the underlying mechanism is that the NRPS A-domain, responsible for recognition of amino acids, has relaxed substrate specificity (Figure 3) (Han et al., 2012).

#### Polymyxins

Polymyxins are a family of non-ribosomal lipopeptide antibiotics composed of ten amino acids, a polycationic heptapeptide ring and a fatty acid derivative at the N terminus (Storm et al., 1977). They are produced by Gram positive bacteria and target Gram negative species, by altering the structure of the cell membrane. The polymyxin family includes polymyxins A, B, D, E (colistin), and M (mattacin) (Shaheen et al., 2011). Polymyxin B exhibits potent antibacterial activity against *Klebsiella pneumoniae*, *Pseudomonas aeruginosa*, and *Acinetobacter* spp. (Gales et al., 2006). However, polymyxins exhibit a remarkable degree of neurotoxicity and nephrotoxicity which limit their clinical use (Li et al., 2006).

In *Paenibacillus polymyxa* PKB1 (the same strain that controls plant fungi by producing fusaricidins, see above), a 40.8 kb polymyxin biosynthetic gene cluster was shown to encode five coding sequences, *pmxA-E*. Three CDS (*pmxA, B, E*) encode subunits of NRPS, each responsible for the modular incorporation of amino acids, while two genes (*pmxC, D*) encode a permease belonging to the ABC-type transporter family (Shaheen et al., 2011). In both *P. polymyxa* PKB1 and *P. polymyxa* E681, the arrangement of the NRPS coding sequences in the *pmx* cluster does not match the amino acid sequence in the produced polymyxin, which is unusual for NRPS-encoded peptides (Choi et al., 2009; Shaheen et al., 2011).

With respect to the diversity of polymyxins, polymyxins differ in the amino acid composition of residues 3, 6, and 7, in the D vs. L stereochemistry of the incorporated amino acids, as well as in the lipid moiety (Choi et al., 2009; Shaheen et al., 2011). In *P. polymyxa* SC2 and *P. polymyxa* PKB1, an allelic variant was uncovered within NRPS domain 3 using bioinformatic analysis of the genome which correlated with incorporation of the D rather than L form of 2,4-diaminobutyrate in amino acid position 3, explaining the mechanism for the production of two subtypes of polymyxin B (Shaheen et al., 2011). With respect to the diversity of residues 6 and 7, *P. polymyxa* E681 and *P. polymyxa* PKB1 produce polymyxins that differ in these amino acids, producing polymyxin A and B, respectively (Shaheen et al., 2011). Bioinformatic analysis revealed that the DNA sequences of the *pmx* gene clusters were 92% conserved at the nucleotide level, but differed considerably in the domains corresponding to modules 6 and 7 (Shaheen et al., 2011). These two sets of observations led the authors to suggest that the diversity of polymyxins arises from mixing and matching of alleles of the NRPS modular domains, hence combinatorial chemistry, rather than relaxed substrate specificity as seen in other secondary metabolites such as fusaricidins (see above).

Another interesting feature of the *pmx* gene clusters is that the polymyxin transporters might also transport fusaricidin, since the *fus* biosynthetic cluster lacks any transporter genes (see above), and as both antibiotics are cationic lipopeptides (Shaheen et al., 2011). The authors found support for this hypothesis, as deletion mutations in *pmxC* and *D* genes also reduced the antifungal activity of fusaricidin against *Leptosphaeria maculans* although the two biosynthetic gene clusters are not linked. It is worth noting that there is no evidence yet of genes responsible for lipidation of the peptide residue in the characterized polymyxin clusters, suggesting that this function might be encoded elsewhere in the genome (Shaheen et al., 2011).

#### Iturins

Iturins are a family of non-ribosomal cyclolipopeptides consisting of seven α-amino acid residues and one ß-amino acid, the latter noted as a unique feature compared to other lipopeptide antibiotics (Constantinescu, 2001; Leclère et al., 2005; Hamdache et al., 2013). The iturin family includes compounds such as bacillomycins D, F and L, bacillopeptins, iturins A, C, E and E, and mycosubtilins (Hamdache et al., 2013). Iturins are produced by different strains of *B. subtilis* and *B. amyloliquefaciens*, and exhibit potent antifungal activity against major phytopathogens including *R. solani, Fusarium oxysporum*, and *F. graminearum*, the latter responsible for Fusarium head blight in wheat (Gueldner et al., 1988; Constantinescu, 2001; Tsuge et al., 2001; Dunlap et al., 2013). The mechanism of action involves disruption of the target fungal plasma membrane (Thimon et al., 1995). In both *B. subtilis* RB14 and *B. amyloliquefaciens* AS43.3, the iturin A biosynthetic operons were shown to contain four coding sequences (*ituDABC*) coding for: a putative malonyl coenzyme A transacylase, a protein with three functions (fatty acid synthetase, amino acid transferase, and peptide synthetase), and two peptide synthetases, respectively (Tsuge et al., 2001; Dunlap et al., 2013).

Regarding diversification within the chemical family, iturin A from *B. subtilis* RB14 has a similar structure as mycosubtilin that is produced by *B. subtilis* ATCC 6633 but with inverted amino acids at the 6th and 7th positions (Tsuge et al., 2001). By comparative analysis of orthologous CDS between these two strains (*ituC* and *mycC*, respectively), it was suggested that the NRPS amino acid adenylation domain may have been intragenically swapped during evolution, which would also imply a HGT event (Tsuge et al., 2001). Comparative genome analysis between at least three sequenced *itu* clusters may reveal further information concerning the diversification of the iturin family (Tsuge et al., 2001; Blom et al., 2012; Dunlap et al., 2013).

#### Bacilysin

Bacilysin is a non-ribosomally produced dipeptide composed of an L-alanine residue at the N terminus and a non-proteinogenic amino acid, L-anticapsin, at the C terminus (Walker and Abraham, 1970; Stein, 2005). Compared to the more elaborate non-ribosomal peptides noted above, bacilysin is noteworthy because it is amongst the simplest peptides in nature, adding to the structural diversity of observed non-ribosomal peptides. Bacilysin is produced by *Bacillus* species such as *B. pumilus, B. amyloliquefaciens*, and *B. subtilis* (Leoffler et al., 1986; Phister et al., 2004) and shown to have antimicrobial activity against various bacteria and fungi such as *Candida albicans* (Kenig and Abraham, 1976). Mechanistically, bacilysin is a prodrug that is activated by the action of a peptidase enzyme that releases the active moiety, anticapsin (Rajavel et al., 2009). Anticapsin inhibits bacterial peptidoglycan or fungal protein biosynthesis through blockage of glucosamine synthetase, resulting in cell lysis (Kenig et al., 1976). Biosynthesis of bacilysin originates from the prephenate aromatic amino acid pathway (Hilton et al., 1988; Parker and Walsh, 2012).

In *B. subtilis* the biosynthesis of bacilysin is encoded by the operon, *bacABCDE* (*ywfB-G*), in addition to a monocistronic gene (*ywfH*) (Inaoka et al., 2003). *bacABC* is likely responsible for the biosynthesis of anticapsin while *bacDE* (*ywfEF*) encodes a ligase and an efflux transporter protein for self protection, respectively (Steinborn et al., 2005; Rajavel et al., 2009). The bacilysin biosynthetic operon is positively regulated by QS pheromones, in particular PhrC (Yazgan et al., 2001; Köroğlu et al., 2011) and negatively regulated by ScoC, a transition state regulator (Inaoka et al., 2009). The transition state in bacteria is a period of decision making.

### Terpenoids

**Figure 4:**
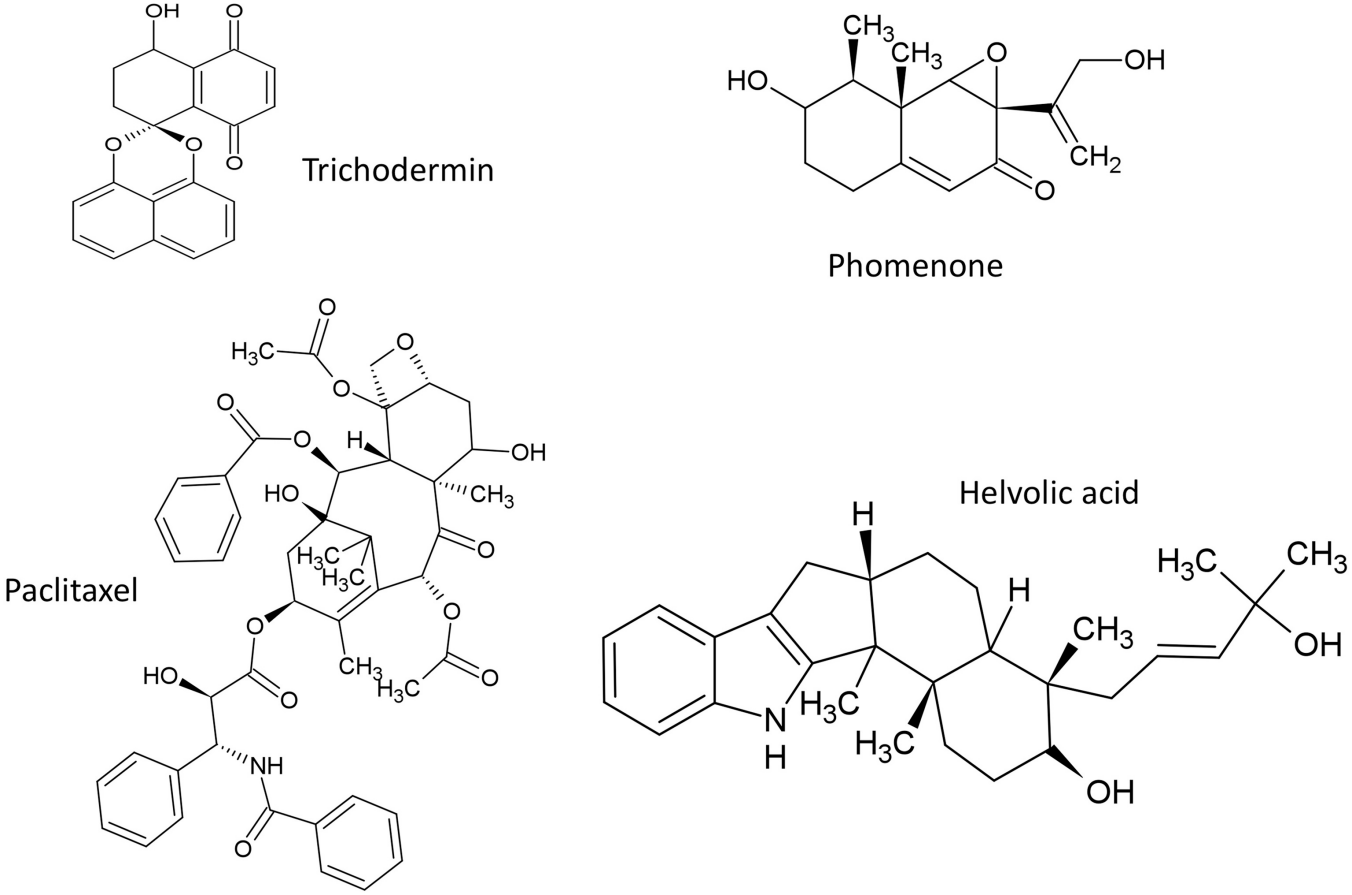
**Structures of terpenoid compounds featured in this review**.

The structures of terpenoids described in this review are summarized (Figure 4).

#### Trichodermin and harzianum A

Trichothecene mycotoxins are produced by some fungal genera such as deoxynivalenol (DON) from *Fusarium*, and harzianum and trichodermin from *Trichoderma arundinaceum and T. brevicompactum*, respectively (Cardoza et al., 2011). Trichodermin was reported to have antifungal activity against the fungal pathogens *Rhizoctonia solani and Alternaria solani* (Chen et al., 2007) as well as other fungal genera (Tijerino et al., 2011). Trichodermin inhibits protein synthesis in eukaryotes by inhibiting peptidyl transferase that catalyzes translational elongation and/or termination (Wei et al., 1974) and by inhibiting peptide-bond formation at the initiation stage of translation (Carter et al., 1976).

**Figure 5:**
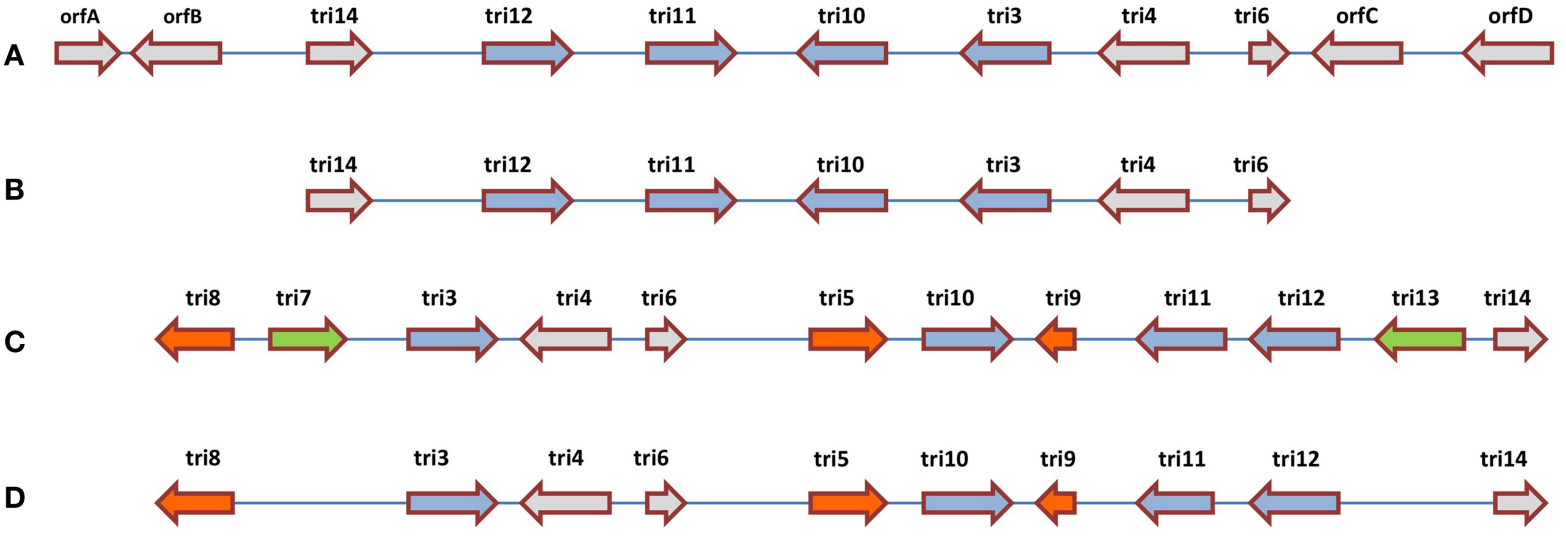
**Comparative analysis of the trichothecene biosynthetic gene clusters in (A) *Trichoderma arundinaceum*, (B) *T. brevicompactum*, (C) *Fusarium sporotrichioides*, and (D) *F. graminearum***. The illustration suggests that the ancestral gene cluster underwent multiple evolutionary events including re-arrangements (blue arrows), gene gain or loss within the same genus (green arrows) and gene gain or loss between genera (orange and green arrows) (adapted from Cardoza et al., 2011).

Comparative analysis has been conducted on the CDS responsible for trichothecene biosynthesis in *Fusarium* and *Trichoderma. In Fusarium*, trichothecenes are encoded by a gene cluster called the TRI cluster; this cluster also encodes regulatory and transport proteins (Proctor et al., 2009). In *Trichoderma*, an orthologous TRI cluster was discovered in which 7 CDS were conserved with *Fusarium*, but the two clusters showed interesting evolutionary divergence (Cardoza et al., 2011) which may be informative for understanding the genetics underlying other anti-fungal metabolites. In *Fusarium*, the TRI cluster includes *tri5* that encodes trichodiene synthase, the first committed step in trichothecene biosynthesis, which catalyzes the cyclization of farnesyl pyrophosphate to form trichodiene (Hohn and Beremand, 1989). In *Fusarium*, *tri5 is* located within the TRI cluster, but surprisingly it is not associated with the orthologous cluster in *Trichoderma*. Three additional CDS responsible for trichothecene biosynthesis in *Fusarium* (*tri7*, *tri8*, *tri13*) are missing from the *Trichoderma* cluster, along with an CDS of unknown function (*tri9*) (Cardoza et al., 2011). Interestingly, two of the apparently conserved biosynthetic CDS (*tri4* and *tri11*, based on sequence homology) were demonstrated to have diverged functionally between *Trichoderma* and *Fusarium* based on heterologous expression analysis: in *Trichoderma*, *tri4* catalyzes three out of four oxygenation reactions carried out by its corresponding *Fusarium* ortholog; *tri11* catalyzes distinctive hydroxylation reactions in *Fusarium* (C-15) and *Trichoderma* (C-4). Finally, amongst the CDS which are conserved between *Fusarium* and *Trichoderma*, head-to-tail vs. head-to-head rearrangements are observed (e.g., *tri3*, *tri4*) (Cardoza et al., 2011). These results demonstrate multiple evolutionary events (rearrangement, functional diversification, gene loss, gene gain) within one biosynthetic gene cluster (Figure 5).

#### Phomenone

Phomenone is a sesquiterpene synthesized by various fungi including *Xylaria* sp., an endophytic fungus isolated from *Piper aduncum*, and reported to have antifungal activity against the pathogen *Cladosporium cladosporioides* (Silva et al., 2010). Phomenone is structurally similar to the PR toxin metabolite of *Penicillium roqueforti* which functions by inhibiting RNA polymerase and thus inhibits protein synthesis at the initiation and elongation steps (Moule et al., 1976). A biosynthetic precursor for phomenone A is aristolochene (Proctor and Hohn, 1993). In *P. roqueforti* NRRL 849, a gene required for aristolochene (*aril*) biosynthesis was characterized and shown to encode a sesquiterpene cyclase named aristolochene synthase (AS) (Proctor and Hohn, 1993). Expression of *aril* occurs in stationary phase cultures and is regulated transcriptionally (Proctor and Hohn, 1993).

#### Paclitaxel (taxol)

The diterpene paclitaxel (Taxol) is reported to be produced by at least 20 diverse fungal endophyte genera inhabiting various plant species (Zhou et al., 2010). Taxol was reported to be produced by some fungal endophytes that inhabit conifer wood and its ecological function was suggested to be a fungicide against host pathogens (Soliman et al., 2013). Taxol acts by stabilizing microtubules and inhibiting spindle function leading to disruptions in normal cell division (Horwitz, 1994). However, Taxol was originally purified from *Taxus* trees (Wani et al., 1971) and shown to be encoded by plant nuclear genes, apparently redundantly. As the number of plant genera that produce Taxol is very few, it is interesting to speculate whether its biosynthetic genes may have been transferred horizontally from fungi to plants.

The Taxol biosynthetic pathway in plants requires 19 enzymatic steps. The first committed step in biosynthesis of plant Taxol is cyclization of GGDP to taxa-(4,5),(11,12)-diene catalyzed by taxadiene synthase (TS) (Hezari et al., 1995). Thirteen plant Taxol biosynthetic genes from *Taxus* were used in BLASTP searches to identify potential homologs in *Penicillium aurantiogriseum* NRRL 62431 (Yang et al., 2014). Seven putative homologous genes were identified though the homology scores were as low as 19%; these genes were claimed to encode: phenylalanine aminomutase (PAM), geranylgeranyl diphosphate synthase (GGPPS), taxane 5α-hydroxylase (T5OH), taxane 13α-hydroxylase (T13OH), taxane 7β-hydroxylase (T7OH), taxane2α-hydroxylase (T2OH) and taxane 10β-hydroxylase (T10OH). Another gene encoding an AT (PAU_P11263) was identified by using BLASTP against the GenBank database. However, no homologs were identified to plant TS; the authors claimed that the fungus might catalyze taxadiene synthesis by a unique enzymatic system (Yang et al., 2014). Position-Specific Iterative BLAST showed one gene from the bacterial genus *Mycobacterium* with potential similarity to plant TS suggesting lateral gene transfer from plants to mycobacteria (Yang et al., 2014).

In a parallel study to isolate fungal Taxol biosynthetic genes, a different approach was taken where PCR primers designed from the plant genes that encode Taxol were used as a primary screen against fungi (Xiong et al., 2013). The study identified putative homologs of fungal TS as well as BAPT (which encodes the critical C-13 phenylpropanoid side-chain CoA acyltransferase) with ~40% sequence identities to their plant counterparts. Despite this progress, other reports remain skeptical that fungi actually encode Taxol (Heinig et al., 2013).

Recent studies have demonstrated complex three-way interactions in Taxol biosynthesis between a Taxol-producing fungal endophyte, other endophytes and the host plant. Host endophytic fungi appear to elicit plant TS transcription or transcript accumulation. Specifically, TS transcript and the corresponding protein were reduced upon treating both young plantlets and old *Taxus* wood with fungicide (Soliman et al., 2013). In a parallel study, co-culture of the Taxol-producing endophyte *Paraconiothyrium* SSM001 with two presumptive fungal endophytes of the same yew tree host elicited paclitaxel accumulation from the endophyte, suggesting inter-species interactions between endophytes inhabiting the same host niche (Soliman and Raizada, 2013).

#### Helvolic acid

Helvolic acid is a fusidane triterpene produced by *Aspergillus fumigatus* (Lodeiro et al., 2009) and the yeast, *Pichia guilliermondii* Ppf9 (Zhao et al., 2010). Helvolic acid was reported to inhibit the spore germination of *Magnaporthe oryzae*, the causal agent of rice blast disease (Zhao et al., 2010). The biosynthetic genes for helvolic acid are clustered as nine genes coding for protostadienol synthase which catalyzes the precursor (17Z)-protosta-17(20),24-dien-3-ol, along with genes that encode squalene-hopene cyclase, four cytochrome P450 monooxygenases, short chain dehydrogenase, two transferases and 3-ketosteroid 1-dehydrogenase (Lodeiro et al., 2009). The authors reported that the P450 monooxygenases from different fungi shared substantial sequence identity across recent evolution, while the transferases duplicated and diversified into paralogous gene families (Lodeiro et al., 2009). This observation suggests that even within a single gene cluster, there may be different selection pressures on adjacent genes belonging to the same biosynthetic pathway. Interestingly, the helvolic acid biosynthetic gene cluster in *A. fumigates* is located in the sub-telomere chromosome region (Lodeiro et al., 2009) which is associated with high rates of evolutionary recombination and diversification. However, the gene cluster lacks introns which is a trait sometimes associated with subtelomeric regions, but this observation might also be evidence of HGT from bacteria (Lodeiro et al., 2009).

### Alkaloids

**Figure 6:**
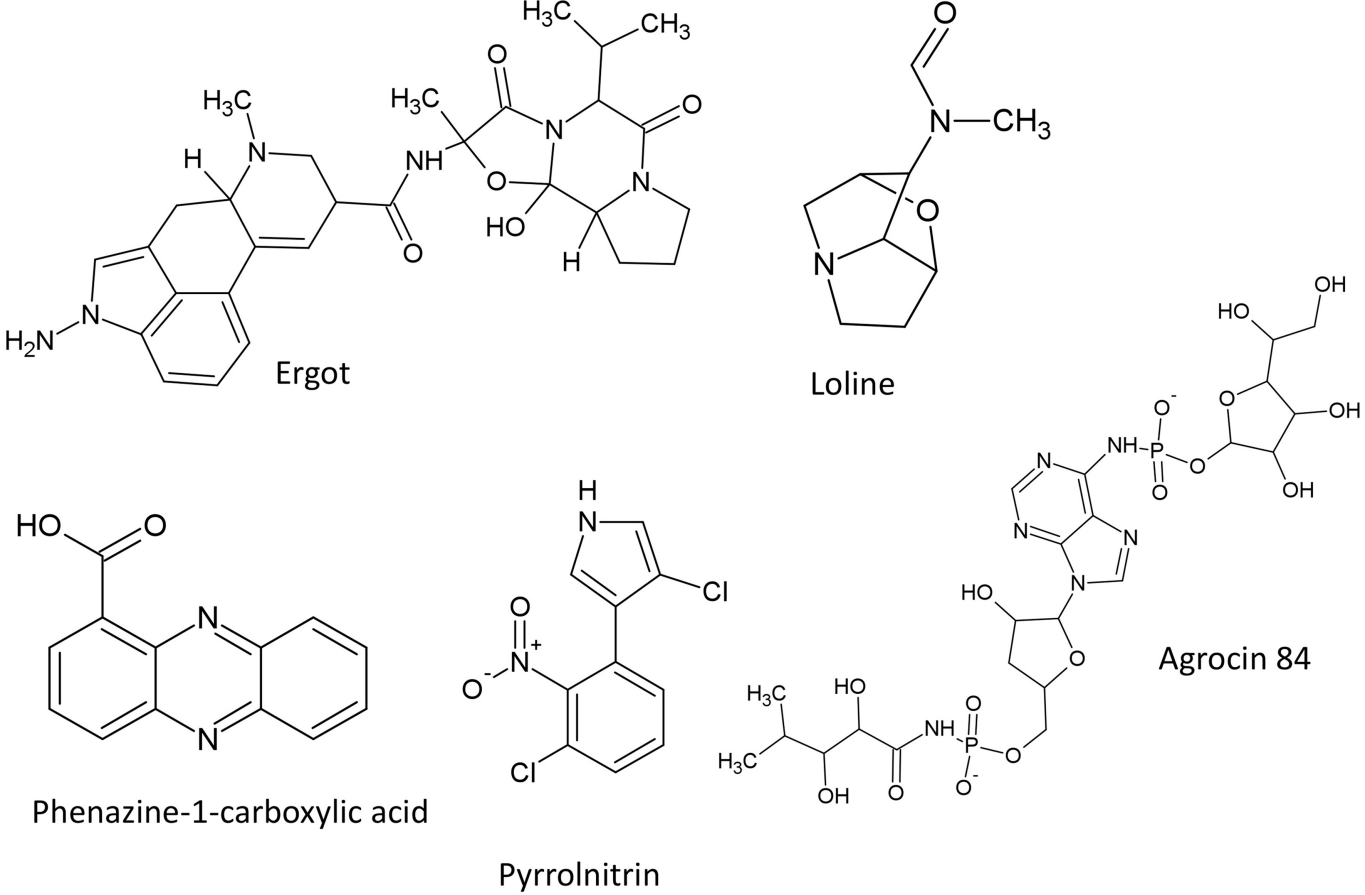
**Structures of featured alkaloids, heterocyclic nitrogenous compounds and bacteriocin**.

The structures of alkaloids described in this review are summarized (Figure 6).

#### Ergot

Ergot alkaloids are produced from the sexual *Epichloe* fungi and their asexual derivatives *Neotyphodium* within the Clavicipitaceae family which inhabit Pooideae grasses (Schardl, 2010). Ergot alkaloids can interact with receptors of the central nervous system and exhibit toxic effect on nematodes, insects, and mammalian herbivores including livestock which graze these grasses (de Groot et al., 1998; Gröger and Floss, 1998). In Europe in the Middle Ages, consumption of ergot-infected grain or grasses caused convulsions, paranoia and hallucinations in livestock and humans, known as St. Anthony's Fire (Dotz, 1980). The diverse ergot alkaloids share a tetracyclic ergoline backbone derived from tryptophan and dimethylallyl diphosphate (Flieger et al., 1997). Gene clusters for ergot alkaloid biosynthesis have been identified in various Ascomycete species belonging to *Aspergillus*, *Penicillium*, and *Claviceps*. Seven genes encode the ergoline scaffold including dimethylallyltryptophan synthase (DMATS) which catalyzes the first committed step. DMATS is responsible for the prenylation of L-tryptophan with dimethylallylpyrophosphate (DMAPP) to produce 4-dimethylallyltryptophan (4-DMAT) (Heinstein et al., 1971). Ergots have diversified into three classes, caused by diverse substituents attached to the carboxyl group of the tetracyclic ergoline backbone, in particular the presence of an amide group (creating ergoamides), a peptide-like amide moiety (creating ergopeptines) or the absence of these moieties (creating clavine alkaloids) (Wallwey and Li, 2011). These structural modifications are responsible for the differential physiological and pharmacological effects of the ergot family, that include treatment of postpartem hemorrhage, leukemia, and Parkinson's disease. The genetic basis for ergot diversification into these 3 major classes is associated with the presence or absence of nonribosomal peptide synthases (NRPS) which catalyze the biosynthesis of the peptide moieties on the ergoline backbone (Wallwey and Li, 2011). For example, four NRPS genes are present in *Claviceps purpurea* (which encodes ergopeptines) but absent in *Aspergillus fumigatus* (which produces clavine alkaloids). Inactivation of these genes suggests that two of the NRPS genes (*lpsA* and *lpsB*) are also responsible for synthesis of the ergoamides (Haarmann et al., 2008). Interestingly, further diversification of the peptide moiety within *C. purpurea* has been reported to be caused by fine-scale allelic diversification of the NRPS genes (Haarmann et al., 2005). There is additional evidence to suggest that diversification of the ergot alkaloid gene clusters is associated with DNA transposons and retroelements, which were observed in the cluster encoding ergovaline, an ergot alkaloid from *Epichloe festucae* associated with livestock toxicity (Fleetwood et al., 2007). As an interesting note, the genes encoding ergovaline were highly expressed only during biotrophic growth of the fungus within the host grass plant not when the mycelia were cultured *in vitro*, suggesting that the host might have a regulatory role in the expression of the fungal gene cluster (Fleetwood et al., 2007).

#### Loline alkaloid

Loline is an indole alkaloid produced by *Neotyphodium uncinatum* fungus, the asexual mutualistic derivative of *Epichloe*, which is known to protect its host plants from insects (Blankenship et al., 2001; Schardl, 2010). The loline biosynthetic pathway was suggested to involve proline and homoserine (Spiering et al., 2005). In *N. uncinatum*, two homologous gene clusters encoding loline were identified, named LOL-1 and LOL-2 (Spiering et al., 2005). The cluster LOL-1 involves nine genes-(*lolF-1, lolC-1, lolD-1, lolO-1, lolA-1, lolU-1, lolP-1, lolT-1, lolE-1*) within a 25-kb chromosomal segment, while the LOL-2 cluster contains the same homologs (except for *lolF*) ordered and oriented the same as in LOL-1. This evidence suggests that the loline clusters may represent a recent segmental duplication event (Spiering et al., 2005).

An interesting ecological situation exists in grasses infected with *Epichloe* fungi (sexual form of *Neotyphodium*): the fungus reduces the ability of these plants to propagate sexually (they choke the inflorescences), which, without compensatory mechanisms, would prevent vertical transmission of the fungus (Zhang et al., 2010). However, to compensate, the fungal stromata attract fly vectors which transmit the fungal spores to other plants, permitting horizontal transfer of the fungus. Loline accumulates in young tissues of the grasses, providing insect protection to these young hosts; however if loline was also to accumulate in the grass inflorescences, it would kill the fly vector of the fungus. Upon further investigation, this apparent paradox was resolved: in these grass inflorescences, transcription of the loline biosynthesis genes was dramatically downregulated compared to plants with healthy inflorescences (infected with the symbiotic asexual *Neotyphodium*), permitting the fly vectors to survive (Zhang et al., 2010). These observations suggest strong selection pressure to evolve the regulatory elements of these genes.

### Heterocyclic nitrogenous compounds

The structures of heterocyclic nitrogenous compounds described in this review are summarized (Figure 6).

#### Phenazines

Phenazines are a group of naturally occurring heterocyclic nitrogenous antibiotics produced exclusively by bacteria and widely reported in fluorescent *Pseudomonas* (Mavrodi et al., 2006, 2013). Phenazines are potent antifungal compounds that can combat soil borne pathogens (Ligon et al., 2000) such as *Rhizoctonia solani, Gaeumannomyces graminis* var. *tritici, Pythium* spp. (Gurusiddaiah et al., 1986) and *Fusarium oxysporum* (Anjaiah et al., 1998). Mechanisms of action include: (1) accumulation of toxic molecules such as hydrogen peroxide and superoxide due to the redox potential of phenazine (Hassan and Fridovich, 1980; Hassett et al., 1995); and (2) elicitation of induced host resistance (Audenaert et al., 2002). Ecologically, the evidence suggests that the plant rhizosphere promotes phenazine-producing bacteria to combat pathogens (Mazzola et al., 1992; Mavrodi et al., 2013).

Phenazine is derived from the shikimic acid pathway, with amino-2-deoxyisochorismic acid (ADIC) as the branchpoint to phenazine (McDonald et al., 2001). ADIC is then converted to trans-2, 3-dihydro-3-hydroxy anthranilic acid which undergoes dimerization to form phenazine-1-carboxylic acid, the first derivative of the phenazines (McDonald et al., 2001). Phenazine biosynthesis in *Pseudomonas fluorescens* is encoded by a single or duplicated core of five CDS, *phzADEFG*, that encode ketosteroid isomerase, isochorismatase, anthranilate synthase, trans-2,3-dihydro-3-hydroxyanthranilate isomerase, and pyridoxamine oxidase respectively (Mavrodi et al., 2013). In *Pseudomonas*, the core may include other CDS such as *phzB* which was duplicated from *phzA*, and *phzC* which encodes 3-deoxy-D-arabino-heptulosonate-7-phosphate synthase that is responsible for diverting carbon from the shikimate pathway to phenazine (Pierson and Pierson, 2010).

Comparisons between *Pseudomomas* species and other genera have revealed conservation yet diversity of the core phenazine biosynthetic CDS. For example, the phenazine biosynthesis operon in *Burkholderia cepacia* maintains the five core enzymes observed in *Pseudomonas* as reviewed (Mavrodi et al., 2006). However, there is evidence to suggest that these coding sequences spread to enteric bacteria and *Burkholderia* species via horizontal gene transfer, because these genes can be observed in plasmids and transposons (Mavrodi et al., 2010). For example, in *Erwinia herbicola*, a biosynthetic cluster of 16 CDS (*ehp*) was isolated from a plasmid, of which 15 coded for D-alanyl griseoluteic acid (AGA) while *ehpR* was observed to encode for resistance to AGA (Giddens et al., 2002). Other differences in the core have also been observed between *Pseudomonas* species and others; for example in both *Burkholderia cepacia* and *Erwinia herbicola, phzA* is not duplicated (Mavrodi et al., 2006).

Structural diversity of phenazines in different species is achieved by specific genes that may be located within the cluster or elsewhere in the genome. For example, in *P. chlororaphi*, the *phzH* gene is located downstream of the phenazine operon, where it encodes an aminotransferase responsible for converting phenazine-1-carboxylic acid (PCA) to phenazine-1-carboxamide, the characteristic green pigment of *P. chlororaphis* (Chin-A-Woeng et al., 1998). In *P. aureofaciens, phzO* was identified as the gene that encodes an aromatic monooxygenase, responsible for catalyzing the hydroxylation of PCA to form the broad spectrum antifungal compound, 2-OH-PCA (Delaney et al., 2001). In *P. aeruginosa* (PAO1), two diversification genes were discovered: *phzM* was shown to be involved in the synthesis of pyocyanin while *phzS* gene encodes a monooxygenase that catalyzes the production of 1-hydroxy phenazine (Mavrodi et al., 2001).

#### Pyrrolnitrin

Pyrrolnitrin is a chlorinated phenylpyrrole antibiotic purified initially from *Burkholderia pyrrocinia* (Arima et al., 1964) then subsequently from other species including pseudomonads, *Myxococcus fulvus, Enterobacter agglomerans*, and *Serratia* sp (Chernin et al., 1996; Kirner et al., 1998; Hammer et al., 1999). Pyrrolnitrin was initially used for treatment of skin mycoses caused by *Trichophyton* fungus, then was developed as an effective fungicide for crops against *Botrytis cinerea* (Hammer et al., 1993), *Rhizoctonia solani* (El-Banna and Winkelmann, 1998) and *Gaeumannomyces graminis* var. *tritici* (Tazawa et al., 2000). In *P. fluorescens*, the pyrrolnitrin biosynthetic operon consists of four coding sequences (*prnABCD*) coding for tryptophan halogenase (*prnA*), a decarboxylase (*prnB*), monodechloroaminopyrrolnitrin halogenase (*prnC*), and an oxidase (*prnD*) (Hammer et al., 1997; Kirner et al., 1998). Comparative analysis indicates that the pyrrolnitrin biosynthetic operon is differentially conserved between divergent species with 59% similarity among diverse bacterial strains such as *Pseudomonas*, *Myxococcus fulvus*, and *Burkholderia cepacia*, with lower similarity shown for *prnA* in *M. fulvus* (45%) (Hammer et al., 1999). Furthermore, RFLP-based polymorphisms within a 786 bp *prnD* fragment suggested that there may have been lateral gene transfer of the *prn* operon from *Pseudomonas* to *Burkholderia pyrrocinia* (Souza and Raaijmakers, 2003). Consistent with such mobility, transposase-encoding genes surrounding the *prn* biosynthetic operon were observed in *Burkholderia pseudomallei* (Costa et al., 2009).

### Volatile compounds

In this section, only the most well studied volatile compound, hydrogen cyanide, is discussed.

#### Hydrogen cyanide (HCN)

Hydrogen cyanide (HCN) is a volatile secondary metabolite produced by *P. aeruginosa*, and diverse rhizosophere fluorescent pseudomonads, where they exhibit biocontrol activity against pathogenic fungi such as *Thielaviopsis basicola*, the fungal causal agent of black root rot of tobacco (Voisard et al., 1989, 1994; Frapolli et al., 2012). Mechanistically, HCN functions by inhibiting important metalloenzymes such as cytochrome *c* oxidase (Blumer and Haas, 2000) and/or by complexing metals in the soil (Brandl et al., 2008). HCN is biosynthesized from glycine (Castric, 1977) in an oxidative reaction catalyzed by HCN synthase, a membrane-bound flavoenzyme (Castric, 1994; Blumer and Haas, 2000). The biosynthesis of HCN occurs in the presence of an electron acceptor such as phenazine methosulfate (Wissing, 1974).

In *P. aeruginosa* PAO1, the HCN synthase biosynthetic operon *hcnABC* was characterized (Pessi and Haas, 2000). *hcnA* was reported to encode a protein similar to formate dehydrogenase while *hcnB* and *hcnC* encode products with similarity to amino acid oxidases (Laville et al., 1998; Svercel et al., 2007). In a phylogenetic analysis of 30 fluorescent pseudomonads, no evidence was found for HGT of the *hcn* gene cluster, but rather that the locus appears to be exclusively inherited vertically (Frapolli et al., 2012). HCN has also been detected in *Chromobacterium violaceum* but the underlying genes have not been reported which might otherwise give new insights into HCN biosynthesis outside of the pseudomonads (Blom et al., 2011).

### Bacteriocin

In this section, only the most well studied compound from this class is discussed. The structure of agrocin 84 described in this review is included (Figure 6).

#### Agrocin 84

Agrocin 84 is a 6-N-phosphoramidate of an adenine nucleotide analog (Roberts and Tate, 1977). This compound is produced by non-pathogenic strains of *Agrobacterium radiobacter* to biocontrol crown gall, a tumorous disease resulting from overproduction of auxin and cytokinin hormones stimulated by the Ti plasmid after it has transferred from *A. radiobacter* and integrated within host plant chromosomal DNA (Wang et al., 1994). Recently, it was shown that agrocin 84 employs a novel mechanism to inhibit leucyl-tRNA synthetases and hence inhibit translation (Chopra et al., 2013), though it was earlier suggested that agrocin 84 acts by inhibiting DNA synthesis (Das et al., 1978).

In *Agrobacterium radiobacter* K84, the biosynthesis and immunity to agrocin 84 is encoded by 17 coding sequences (the *agn* operon) located on a 44-kb conjugal plasmid, *pAgK84*, though the plasmid has 36 CDS in total (Kim et al., 2006). The two most interesting CDS are *agnB2* and *agnA* which encode aminoacyl tRNA synthetase homologs. The agrocin 84 antibiotic is essentially a nucleotide attached to an amino acid-like moiety (methyl pentanamide), and its mode of action was proposed to involve competitive binding to the active site of leucyl-tRNA synthetases (Reader et al., 2005). *agnB2* encodes a leucyl-tRNA synthetase homolog that confers self-immunity to agrocin 84 since it does not bind the antibiotic (Kim et al., 2006). Normally, a tRNA synthetase acts as a ligase that catalyzes the attachment of an amino acid to a tRNA which includes an anticodon; the catalysis results in a phosphoanhydride bond between the amino acid and ATP as the initial step in the aminoacylation of tRNA (Ibba and Söll, 2000). Surprisingly, *agnA* encodes a truncated homolog of an asparaginyl-tRNA synthetase which lacks the anticodon-binding domain, but maintains the catalytic domain. Thus, *agnA* appears to be a fascinating example of a gene that evolved from an ancient tRNA synthetase (for arginine), but now is a biosynthetic enzyme for an antibiotic that inhibits a paralogous enzyme (for leucine attachment) (Kim et al., 2006).

The *agn* operon may have an evolutionary history of horizontal gene transfer, as *pAgK84* is inter and intra species transferable: *Rhizobium* that received the *pAgK84* plasmid from *Agrobacterium* as trans-conjugates could synthesize agrocin 84 and received immunity as well (Farrand et al., 1985).

A final fascinating feature of the *agn* system is an apparent second form of ancient evolutionary pressure on the genes responsible for the biosynthesis of the antibiotic. Agrocin 84 is a chemical mimic of agrocinopines, a class of compounds that is a source of plant-derived nitrogen for the pathogens targeted by the antibiotic; the pathogens have their own Ti plasmids that encode for transporters that not only transport agrocinopines but also agrocin 84 (Ellis and Murphy, 1981; Hayman and Farrand, 1988; Kim and Farrand, 1997). Hence the *agn* biosynthetic genes evolved to create a chemical structure that not only mimics the tRNA synthetase substrate of the pathogen target, but also targets its nitrogen uptake machinery.

### Enzymes

In this section, only the most well studied anti-fungal enzyme, chitinase, is discussed.

#### Chitinase

Chitinases are enzymes that break down chitin, one of the fungal cell wall components composed of repeated units of N-acetyl-D-glucos-2-amine, linked by β-1,4 glycosidic bonds (Bhattacharya et al., 2007). Fungi and hence chitin are enriched in soil and thus soil microbes are abundant sources of chitinases (also to target insects) (Hjort et al., 2010). Examples of chitinase-producing microbes include: fluorescent *Pseudomonas* strains isolated from the sugarcane rhizosphere that can target *Colletotrichum falcatum*, the causative agent of red rot disease in this crop (Viswanathan and Samiyappan, 2001); *Actinoplanes missouriensis* that antagonizes *Plectosporium tabacinum*, the causal agent of lupin root rot in Egypt (El-Tarabily, 2003); and *Stenotrophomonas maltophilia* that suppresses summer patch disease in Kentucky bluegrass (Kobayashi et al., 2002). Chitinases are produced by diverse bacterial genera including *Pseudomonas*, *Streptomyces*, *Bacillus*, and *Burkholderia* (Quecine et al., 2008). Chitinases are divided into two major categories, exochitinases and endochitinases. Of the four reported endochitinase family members (glycoside hydrolase families 18, 19, 23, and 48), primarily families 18 and 19 have been reported in bacteria, with only a single example of a family 23 chitinase (Prakash et al., 2010).

**Figure 7:**
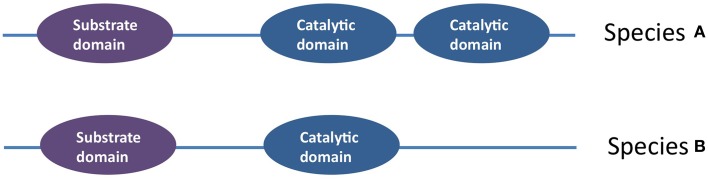
**An example of intra- coding sequences diversification within an anti-microbial gene cluster: amongst the Family 18 chitinases is an example of a chitinase in which the catalytic domain has been duplicated (Howard et al., 2004)**.

In *Stenotrophomonas maltophilia* 34S1, the chitinase family 18 gene has one CDS that encodes for a protein with seven domains: a catalytic domain, a chitin binding domain, three putative binding domains, a fibronectin type III domain and a polycystic kidney disease domain (Kobayashi et al., 2002). Bacterial chitinase family 18 has been shown to display different types of diversity. First, sequence analysis has shown that the catalytic domain and substrate binding domain, which are separated by a linker, have evolved independently. As the domain sequences do not match the taxonomies of their hosts, it has been suggested that domain swapping has been an important generator of diversity in this family, combined with HGT (Figure 7) (Karlsson and Stenlid, 2008). Unusual examples of chitinase genes are those that contain multiple family 18 catalytic domains within the same peptide that appear to function independently of one another (Howard et al., 2004). Additional examples of family 18 biodiversity include genes that contain non-consensus sequences at the catalytic site, as well as a bacterial subgroup that consists solely of a catalytic domain (Karlsson and Stenlid, 2008).

Unlike family 18 chitinases that are widely distributed among the prokaryotes, family 19 chitinases are restricted to green non-sulfur and purple bacteria, as well as actinobacteria (Prakash et al., 2010). Based on sequence alignments of family 19 chitinases in prokaryotes and eukaryotes, strong evidence has emerged that this gene family in actinobacteria and purple bacteria was derived from flowering plants by HGT (Prakash et al., 2010). Furthermore, HGT from plants to purple bacteria may have occurred as two independent events in the distant past, followed more recently by HGT to actinobacteria (Prakash et al., 2010). The core architecture and catalytic sites of bacterial and plant family 19 chitinases are nearly identical. The sequence analysis further suggests that there was subsequent HGT from purple bacteria and actinobacteria to nematodes and arthropods, respectively (Prakash et al., 2010).

## Discussion

The objective of this paper was to review the biodiversity of anti-microbial compounds, their mode(s) of action and underlying biosynthetic genes within plant associated microbes. This review covered diverse biosynthetic gene clusters that encode polyketides, non-ribosomal peptides, terpenoids, alkaloids, heterocyclic nitrogenous compounds, volatile compounds, bacteriocins, and lytic enzymes. The reviewed evidence suggests that these biosynthetic genes have diversified at different orders, each based on distinct evolutionary mechanisms:

### Species level diversification

**Figure 8:**
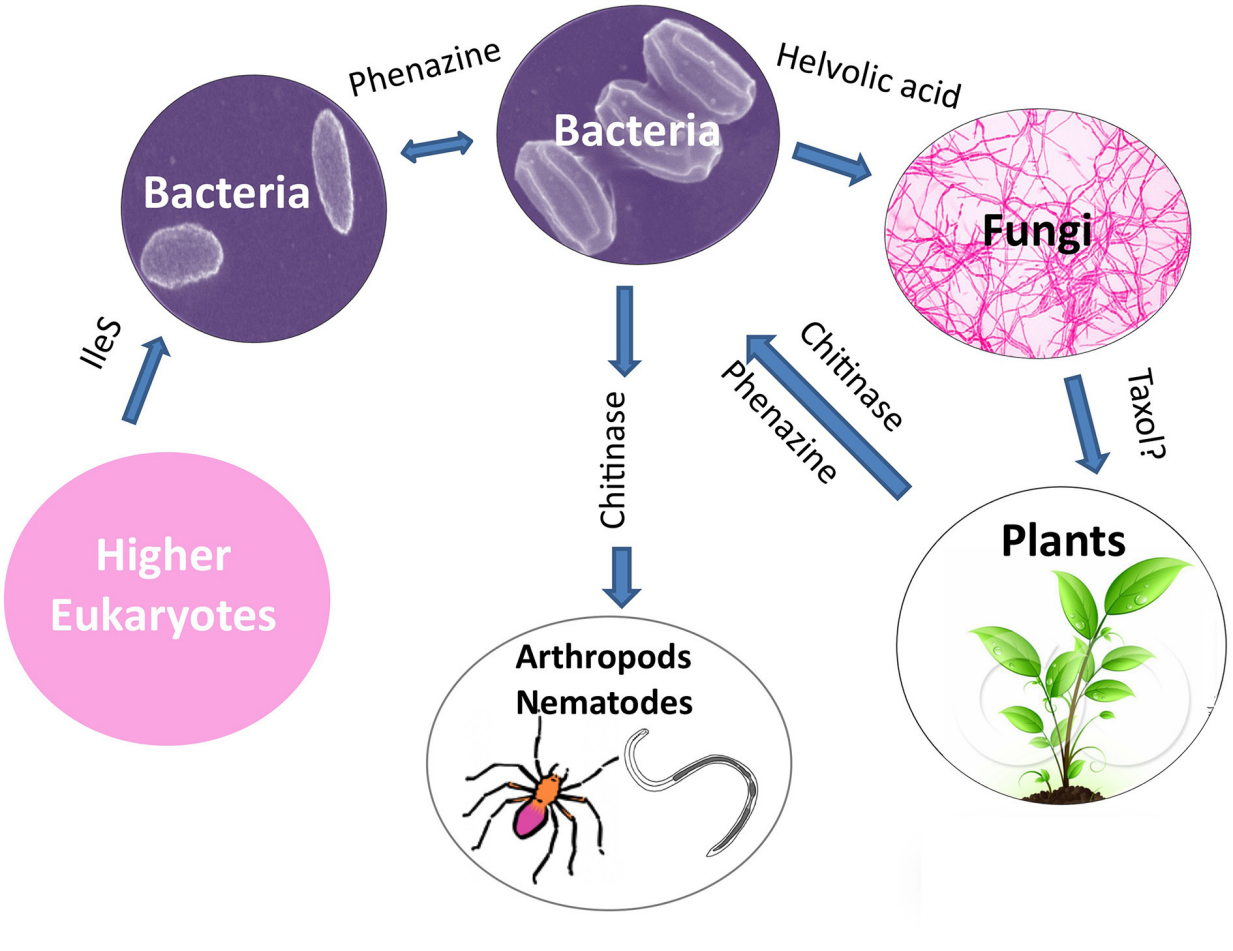
**Potential examples of horizontal gene transfer of anti-microbial gene clusters leading to species level evolutionary diversification**.

An emerging theme from the literature is that horizontal gene transfer (HGT) appears to have played a major role in the evolutionary diversification of plant-associated microbial species through inheritance of anti-microbial traits. There is evidence that HGT may have occurred from: bacteria to bacteria such as those that inhabit the rhizosphere (e.g., phenazine); from bacteria to fungi (e.g., helvolic acid); from bacteria to nematodes and arthropods (e.g., chitinase family 19); possibly from fungi to plants (e.g., Taxol); from plants to bacteria (e.g., phenazine and chitinase family 19); and even from higher eukaryotes to bacteria (e.g., IleS, pseudomonic acid resistance protein) (Figure 8). As noted in the literature, diverse factors might have facilitated these remarkable gene transfer events including: (1) the clustering of genes encoding the secondary metabolite; (2) homologous recombination between chromosomes and trans-conjugated plasmids (e.g., phenazine, zwittermicin A); (3) the presence of mobile elements (DNA transposons and retroelements) flanking the biosynthetic operons (e.g., zwittermicin A, phenazine and ergovaline); and (4) the presence of genes that encode self-immunity to the antibiotic within the biosynthetic cluster as otherwise receiving the cluster would have caused immediate suicide (e.g., mupirocin). It is worth noting that some gene clusters show no evidence of HGT (e.g., hydrogen cyanide).

### Genome level diversification

A second emerging theme from the literature is that a subset of associated plant- associated microbial genomes have diversified with respect to duplications of entire gene clusters responsible for the synthesis of antimicrobial compounds. For example, as noted above, in *Neotyphodium uncinatum*, there are two homologous gene clusters that encode loline, LOL-1 and LOL-2, the likely result of a segmental duplication event within this fungus. Another noted example is from *Bacillus* species in which three tandemly duplicated gene clusters, *pks1*, *pks2*, and *pks3*, encode the polyketides, bacillaene, macrolactin, and difficidin, respectively, the likely result of a homologous recombination event.

### Intra gene cluster diversification

A third interesting theme from the literature is that gene clusters encoding anti-microbial compounds have extensively diversified within, to permit biochemical diversification. The biosynthetic genes for these compounds are clustered in fungi or organized into operons in bacteria—in the latter, they are generally located on chromosomes but occasionally on plasmids (e.g., agrocin 84). Diversity within each cluster can include varying combinations of biosynthetic coding sequences (CDS), transporters for the respective compound, regulatory genes and CDS that confer self-immunity (e.g., mupirocin, agrocin 84). The biosynthetic operons vary in how many CDS synthesize the core skeleton (e.g., synthetases) as well as in how many encode decoration enzymes (e.g., hydroxylases, acyltransferases). However, the decoration enzymes may be encoded outside the gene cluster (e.g., phenazine operon). Furthermore, the biosynthetic CDS may be organized into genetic modules (e.g., NRPS) that vary in number. Each gene cluster is also associated with distinct DNA regulatory elements, for example to receive signals such as from quorum sensing. For example, comparative analysis of the trichothecene biosynthetic gene clusters (TRI) in *Fusarium* and *Trichoderma* showed multiple evolutionary diversification events within a single biosynthetic gene cluster family (e.g., head-to-tail vs. head-to-head rearrangements) (Figure 5). In another example, comparative analyses of the polymyxin operon showed mixing and matching of CDS, resulting in diversification of the compounds. Similarly, diversification of the phenazines likely arose through a diversity of biosynthetic decoration enzymes (e.g., hydroxylases). Another intriguing observation is from the helvolic acid biosynthetic gene cluster, in which transferase CDS were shown to have duplicated and diversified into paralogous gene families. As noted above, an interesting feature of this gene cluster is that it located in the sub-telomere chromosome region which is associated with high rates of evolutionary recombination.

### Diversification within coding sequences (CDS)

A final emerging theme from the literature is that diversification of anti-microbial traits in plant-associated microbes arose from allelic diversification. For example, intragenic swapping of domains was observed within the same genetic module (e.g., iturin A, mycosubtilins). As another example, whereas most chitinase genes possess a single catalytic domain, examples were noted where a single CDS encodes two catalytic domains (Figure 7). In general, the literature notes that domains within the same CDS can evolve independently (e.g., catalytic vs. substrate binding domains of chitinase); combined with the existence of linker peptides between domains as sites of homologous recombination, these features can result in novel alleles following domain swapping (e.g., family18 chitinases). Whereas, such allelic diversification plays a major role in the diversification of compound structures, caused for example by DNA mutations within the substrate binding domain, the literature demonstrates examples where biochemical diversity has arisen from relaxed substrate specificity of the biosynthetic enzymes (Figure 3). A representative example of the latter is the promiscuous fusaricidin NRPS in which the same recognition domain in different species can recognize and incorporate different amino acids, and furthermore it can recognize amino acids beyond the 21 standard amino acids translated by the ribosome, which results in significant structural diversity.

### Dynamic evolutionary driven by selection pressures

It is interesting to speculate on the evolutionary selection pressures that have led to the diversification at the various biological levels noted above. At the most basic level, diversification was likely driven by a three-way co-evolution between the plant-associated microbe, its target pathogen and the host plant. This co-evolution may have occurred within a specific plant tissue niche or within soil associated with the rhizosphere (e.g., phenazine and PLt to combat soil-borne pathogens). However, there is also evidence for four-way interactions, to also include additional microbes (e.g., Taxol, jadomycin) and insects (e.g., ergovaline). These complex interactions can be bi-directional (e.g., loline). Within the producing organism, there is evidence for selection pressure to coordinate biosynthesis of the anti-microbial compound with the life cycle of the microbe (e.g., fusaricidins). There may also have been selection for genetic efficiency (e.g., potential sharing of transporter genes between polymyxin and fusaricidin). These selection pressures have led to fascinating individual stories, including the evolution of mimicry to facilitate antibiosis (e.g., agrocin).

## Ecological and evolutionary lessons

**Figure 9:**
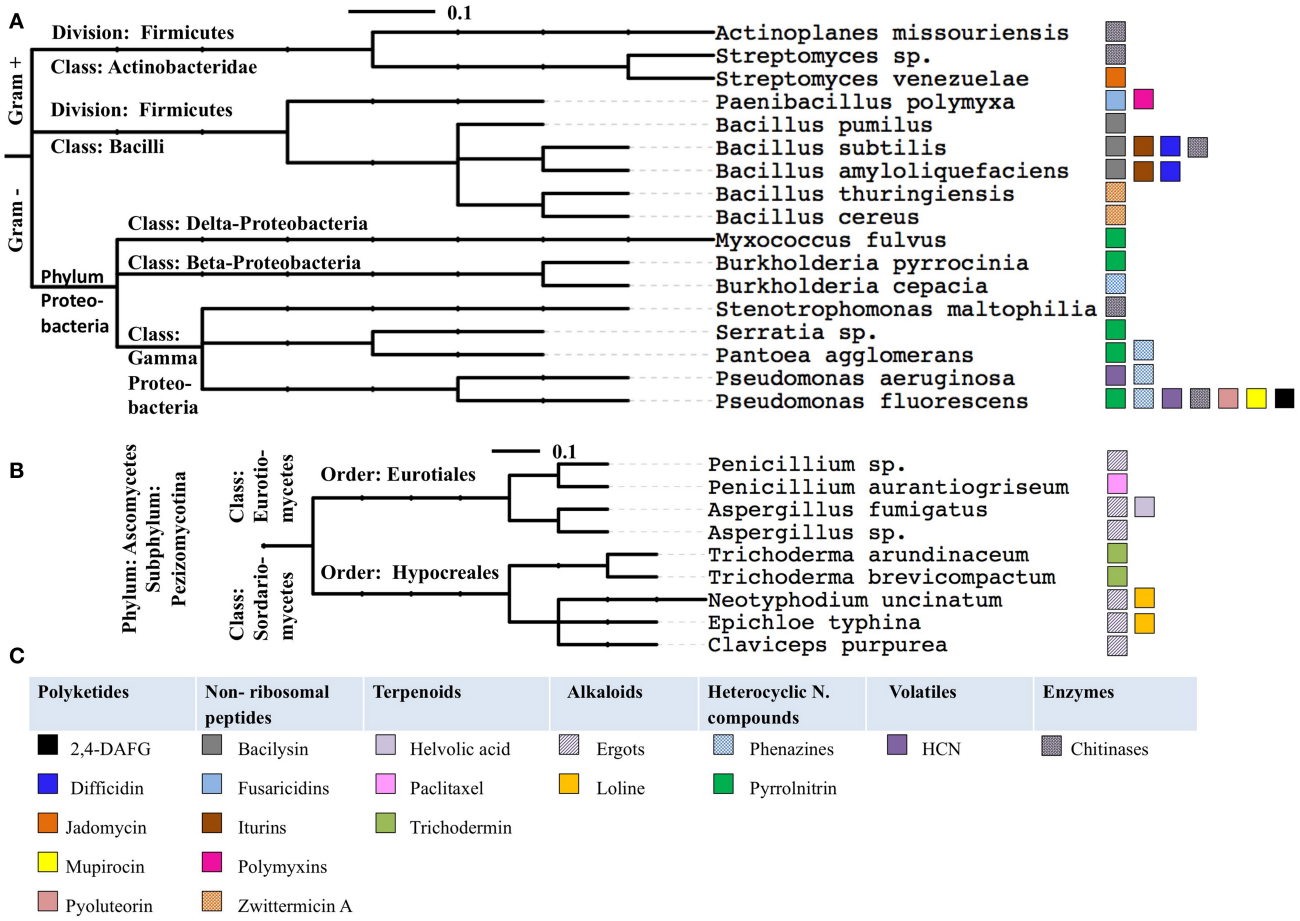
**The anti-microbial compounds reviewed in this study grouped by the phylogeny of their microbial hosts, for bacteria (A) and fungi (B)**. The phylogenetic trees were generated using the interactive Tree of Life website (Letunic and Bork, 2011). The anti-microbial compounds produced by these species color coded, panel of color coded as indicated **(C)**. The scale bar represents the number of nucleotide substitutions per site.

When the examples of anti-microbial pathways were grouped by the phylogeny of their host microbes, several trends were observed (Figure 9, Tables 1, 2). Specifically: (1) some anti-microbial genes are apparently widely distributed among diverse taxonomic classes of bacteria (e.g., chitinases); (2) some metabolic pathways are widely distributed within one taxonomic class such as pyrrolnitrin that shows up in more than half of the presented species of Proteobacteria; (3) other anti-microbial pathways appear to be more restricted (e. g., fusaricidin, polymyxin, jadomycin). These results may correlate to the evolutionary age of these genetic pathways, or may represent a bias based on how well the pathway has been studied. More widespread genome sequencing and/or the use of orthologous gene probes may help to inform the evolutionary origins of these anti-microbial pathways.

### Bacterial pathway lessons

The selected examples of anti-microbial pathways from plant-associated bacteria found in the literature and presented in this review are distributed across Proteobacteria, Actinobacteria and Firmicutes (Figure 9). This may not be surprising as Proteobacteria and Actinobacteria are among the most widespread bacterial taxa associated with plants, perhaps because of their saprophytic capabilities (Bulgarelli et al., 2012).

Within these phyla, *P. fluorescens* (Proteobacteria) and *B. subtilis* (Firmicutes) were observed to produce a plethora of diverse antimicrobial compounds belonging to diverse chemical classes including polyketides, non-ribosomal peptides, heterocyclic nitrogenous compounds, volatiles and enzymes which reflect the diversity of the metabolic machineries of these species. As *P. fluorescens* and *B. subtilis* are both model systems, these results also support the above note of the bias within this literature.

*Bacillus* sp. and *Pseudomonas* sp. are ubiquitous microbes that can survive in diverse ecological niches (Compant et al., 2005). Both have elegant survival strategies that involve the production of antibiotics, surfactin, cyanide, biofilms, and induction of host resistance (Espinosa-Urgel, 2004; Dini-Andreote and van Elsas, 2013). These unique adaptations have led to their widespread study and use as biocontrol agents (Santoyo et al., 2012).

Genome analysis of *P. fluorescens* has provided insight into its ecological competency and evolutionary mechanisms. The versatile and rapid adaptability of *P. fluorescens* to diverse environmental clues may be attributed to over 200 characterized signal transduction proteins which enhance its sensing capability (Garbeva and de Boer, 2009; Humair et al., 2010). With respect to co-evolution, the *P. fluorescens* genome is exceptionally rich in repetitive extragenic palindromic (REP) elements, target sites for transposases and recombinases, with 1052 REP elements identified in *P*. *fluorescens* Pf-*5* (compared to 21 in *P. aeruginosa* PAO1 and 365 in *P. syringae* DC 3000) (Tobes and Pareja, 2005, 2006). REPs likely affected genome evolution either by gene gain, loss or rearrangement (Silby et al., 2011). The latest version of the genome sequence and annotation of *P. fluorescens* was recently released (Martínez-García et al., 2015).

*B. subtilis* is naturally competent genetically, with a cascade of competence-specific DNA-uptake proteins that bind and transport DNA, in addition to a dynamic recombination mechanism which transforms chromosomal or plasmid DNA via different pathways (Chen and Dubnau, 2004; Kidane et al., 2009). Additionally, the *B. subtilis* genome encodes integrative and conjugative element binding (*ICEBs1*) proteins responsible for excision, integration, transfer of DNA (Lee et al., 2007) that likely have facilitated HGT. Comparative genomic analysis of *B. subtilis* strains revealed 298 accessory segments that potentially originated from mobile elements including plasmids, transposons and phages. This implies extensive HGT events that lead to diversification of the arsenal of anti-microbial pathways within *Bacillus* (Zeigler, 2011). The complete genome sequence and genome annotation of *B. subtilis* is available (Barbe et al., 2009; Belda et al., 2013).

### Fungal pathway lessons

In contrast to bacteria, all the fungal examples presented in the review belong to a single classification—the Pezizomycotina (filamentous fungi), a subdivision of Ascomycota, the largest phylum of fungi (Blackwell, 2011) including representatives from Eurotiomycetes and Sordariomycetes (Figure 9). Pezizomycotina has an ancient origin in the Cambrian period, ca 530 Mya (Prieto and Wedin, 2013).

Pezizomycotina species are the most ubiquitous fungi with extremely diverse lifestyles, suggesting a corresponding diversity of ecological strategies (Spatafora et al., 2006; Beimforde et al., 2014) reflected in their production of a range of anti-microbial metabolites. There may be at least two reasons for this metabolic diversity, HGT and recombination. First, HGT from bacteria to fungi was previously reported in Ascomycota, of which 65% were observed in Pezizomycotina (Marcet-Houben and Gabaldón, 2010). Second, secondary metabolism gene clusters in Pezizomycotina show evidence of recent gene expansion (Arvas et al., 2007). Interestingly, most of these genes are located in the sub-telomere region (Rehmeyer et al., 2006) that is associated with a considerable high rate of recombination and correspondingly rapid evolution compared to other regions in the genome (Freitas-Junior et al., 2000), an example represented in this review by helvolic acid (Table 1).

## Gaps and future perspectives

Despite the apparent progress in understanding the genetic mechanisms underlying the diversity of anti-microbial compounds produced by plant-associated microbes, significant gaps and opportunities remain. The major challenge is that a vast majority of plant associated microbes are unculturable, a phenomena that, to a far extent, limits our understanding of species diversification and evolution. It is worth noting that considerable progress toward cultivation of unculturable microbes has started to be achieved (Pham and Kim, 2012; Stewart, 2012). The modified cultivation methods attempt to simulate the natural environment, and include community culturing, and the use of high-throughput microbioreactors and laser microdissection (Pham and Kim, 2012). Another challenge is that the literature appears to be biased for model organisms, with insufficient data from other organisms in the phylogenetic tree for comparative genomics and evolutionary studies. For example, despite our efforts to originally focus this review only on anti-microbial pathways from endophytes, it became clear that the number of associated genes from endophytes has largely been unexplored, compared to free living rhizosphere model species. Indeed, there remains a lack of detailed genetic analysis underlying many anti-microbial compounds across microbes (endophytic and non-endophytic) and a lack of information to connect allelic diversity with compound diversity.

With respect to understanding the biosynthetic pathways of these metabolites, more information is needed as to the extent that diverse anti-microbial pathways coordinate and share biosynthetic enzymes. An important question in metabolic biosynthesis is understanding how chemical substrates are channeled along metabolic pathways from one enzyme to the next; from this review, it appears that some anti-microbial pathways solve this problem by using mega-synthase enzymes (e.g., zwittermicin A), but for other pathways, investigation of enzyme-enzyme interactions will be informative. An interesting future area of study will be to investigate the subcellular location of biosynthetic and storage proteins, especially of self-toxic compounds that may need to be sequestered. To that end, there have been advances in studying compartmentalization and secondary metabolite trafficking machinery (Roze et al., 2011; Lim and Keller, 2014; Kistler and Broz, 2015), which offer strategies to move forward.

A significant challenge in this discipline is the study of anti-microbial compounds in their native ecological context, as most reports are based only on *in vitro* studies. In particular, because the target pathogen affects the host plant, more information is needed as to how the plant and the anti-pathogenic microbe coordinate and regulate one another. For example, in the jadomycin pathway, evidence suggests that the plant sensing of the pathogen stimulates the anti-pathogen pathway in the associated beneficial microbe. The potential complexity of plant-microbe interactions and associated signaling networks are well studied in model systems such as *Rhizobium* (Janczarek et al., 2015). Though *Rhizobium* is a symbiotic microbe of legume plants, these studies suggest that a wealth of information remains to be explored for other plant-associated microbes, in particular endophytes (Kusari et al., 2012).

Also within the ecological context, basic biochemical questions are raised such as whether the anti-microbial pathway is regulated by the target pathogen, for example feedback inhibition once the pathogen has been eliminated. To help understand the genetic regulation of these anti-microbial pathways, analysis of gene expression with respect to the microbial life cycle would be a useful avenue of investigation, similar to the interesting findings from the fusaricidin pathway. An interesting study concerning aflatoxin, a polyketide mycotoxin, revealed strong evidence for the potential link between the fungal growth stage and polyketide biosynthesis (Zhou et al., 2000). Furthermore, intracellular tracking of aflatoxin biosynthetic enzymes in *Aspergillus parasiticus* showed significant accumulation in the vacuoles of specific cells but its absence in neighboring ones (Hong and Linz, 2008). This surprising result led Roze et al. (2011) to hypothesize the possibility of special and temporal gene expression of the associated biosynthetic pathway, at different developmental resolutions ranging from a single cell to fungal colony.

A related major challenge is that there are many natural products that exist in the literature that were initially isolated as part of screens for new compounds from total extracts, and hence the ecological functions of these compounds, as well as their underlying genes, remain unknown.

As anti-pathogenic metabolites may be self-toxic, the evolution of self-resistance is a particularly fascinating avenue of study, which this review demonstrates has been investigated for a limited number of pathways (e.g., mupirocin). Diverse self-resistance mechanisms have been reported in the microbial literature (Schäberle et al., 2011; Westman et al., 2013; Stegmann et al., 2015), suggesting that each plant-asssociated microbe with anti-microbial activity may employ unique self-protection strategies.

The recent advances in genome sequencing combined with gene editing tools will facilitate more in-depth analysis of orthologous biosynthetic genes in diverse species. Bioinformatic genome mining of biosynthetic gene clusters, combined with new advances in metabolomics, may also lead to the discovery of a diverse array of novel bio-active natural products. Moreover, merging these techniques with knowledge of microbial co-evolution and ecology (Vizcaino et al., 2014) along with advanced microscopy and imaging techniques will open a new era of discovery to harvest the diversity of natural products to combat evolving pathogens.

## Author contributions

WM wrote the manuscript, and WM and MR edited the manuscript.

### Conflict of interest statement

The authors declare that the research was conducted in the absence of any commercial or financial relationships that could be construed as a potential conflict of interest.
